# Forecasting short-term data center network traffic load with convolutional neural networks

**DOI:** 10.1371/journal.pone.0191939

**Published:** 2018-02-06

**Authors:** Alberto Mozo, Bruno Ordozgoiti, Sandra Gómez-Canaval

**Affiliations:** Dpto. Sistemas Informáticos, Universidad Politécnica de Madrid, Madrid, Spain; National Chiao Tung University College of Biological Science and Technology, TAIWAN

## Abstract

Efficient resource management in data centers is of central importance to content service providers as 90 percent of the network traffic is expected to go through them in the coming years. In this context we propose the use of convolutional neural networks (CNNs) to forecast short-term changes in the amount of traffic crossing a data center network. This value is an indicator of virtual machine activity and can be utilized to shape the data center infrastructure accordingly. The behaviour of network traffic at the seconds scale is highly chaotic and therefore traditional time-series-analysis approaches such as ARIMA fail to obtain accurate forecasts. We show that our convolutional neural network approach can exploit the non-linear regularities of network traffic, providing significant improvements with respect to the mean absolute and standard deviation of the data, and outperforming ARIMA by an increasingly significant margin as the forecasting granularity is above the 16-second resolution. In order to increase the accuracy of the forecasting model, we exploit the architecture of the CNNs using multiresolution input distributed among separate channels of the first convolutional layer. We validate our approach with an extensive set of experiments using a data set collected at the core network of an Internet Service Provider over a period of 5 months, totalling 70 days of traffic at the one-second resolution.

## Introduction

Nowadays, there is a general consensus in the Information and Communications Technology (ICT) industry regarding the higher demands in network bandwidth and speed that Internet and mobile systems will have to meet in comparison with today’s networks. Additionally, cloud and virtualization technologies are enabling applications and networks to abstract away from their underlying physical infrastructure, and therefore to programmatically provide networking as a service. Analysts predict that more than 90 percent of the Internet traffic will go through data centers in the short term. Therefore, it is of unquestionable interest to cloud providers to research and develop forecasting mechanisms for resource demand prediction for their application to the management of data center infrastructures and resources. Thanks to the dynamicity with which these management tasks can be done today, the amount of resources a cloud provider can devote to the servicing of its customers’ demands can be adjusted in near-real time, depending on their actual needs. If the ability to easily scale up and down network and data center resources is combined with reliable forecasts of demand patterns, service and infrastructure providers could become able to obtain significant savings in energy consumption, as well as to avoid performance degradation due to resource shortages. In this context, a growing trend is to not just react to network changes, but anticipate them as much as possible by forecasting the evolution of network and infrastructure conditions in data centers. Around this topic, considerable research activity has emerged in the last years. In section 1 we present a landscape of the most important works in this research area.

In this context we propose the application of artificial feed forward neural networks (ANNs), and in particular, convolutional neural networks (CNNs) for forecasting short-term changes in network traffic dynamics. Specifically, we want to forecast the number of user sessions (i.e., transport level connections or flows) expected to be crossing a network link in the range of seconds, using as input a time series signal comprised by the previous observations of this value sampled at one-second intervals. From an applicability perspective, the number of user sessions that are crossing the core network of a data center can be considered as an indicator of virtual machine activity. For example, if a network and infrastructure orchestrator in a data center can compute a reliable estimate of the number of user sessions that will be active in the following seconds or minutes, it will be able to shape its infrastructure accordingly (e.g. anticipating decisions for switching on and off physical machines) to avoid the energy inefficiencies and costs incurred by overprovisioning or poor performance due to overwhelmed machines. Virtual machines typically have a startup time of a few seconds to a minute, [1], so anticipating significant load changes in this time scale should be helpful to make placement decisions. To the best of our knowledge the majority of the previously investigated solutions address the long-term prediction of global Internet traffic dynamics, and in particular, no solutions have been proposed for predicting the short-term network dynamics of data centers. The behaviour of network traffic in the short-term scale is highly chaotic, and so far it has been unclear whether any meaningful structure can be exploited to make reliable forecasts. We hypothesize that there may exist meaningful non-linear dependencies between present and past events. Traditional approaches for time series analysis and forecasting, such as ARIMA and GARCH, are not likely to benefit from such dependencies. We therefore propose to use ANNs, and in particular CNNs, because of the ability of these methods to model non-linear relationships in the input data. In addition, CNNs have been revealed successful and efficient in the exploitation of the temporal nature of the data.

In order to improve the quality of the forecasts we propose a combination of three different techniques. First, we adopt a multiresolution strategy, taking as input a vector comprising past observations at different levels of granularity. The aim of this strategy is to increase the amount of contextual information (i.e. past observed values) without compromising the scalability of the model. Second, although the values of the time series we consider are sampled at the one-second frequency, we also explore the problem of producing coarse-grained forecasts (up to the 64 second-resolution) in order to mitigate the amount of noise observed in the signal at the original resolution. Nowadays, actuation subsystems in a data center involve anticipatory decisions in the range of 60–90 seconds and therefore, coarse-grained forecasts up to 64 seconds are directly applicable in such scenarios. Finally, we feed each resolution of the input vector to a different convolutional channel in the CNN. Each channel will thus learn a different set of filters for each input resolution. As these channels are added together, the relationship between different resolutions (i.e. channels) is learned and modelled by the neural network.

In order to validate our proposal, we carried out an extensive and in-depth set of experiments on a real traffic data set collected at the core network of a medium-sized Spanish Internet Service Provider (ISP) over a period of three years. This network processes around 200,000 packets per second, which we aggregated and transformed into a time series representing the number of active user sessions (i.e., TCP connections) per second. We selected and analysed five months of data, and kept two consecutive weeks of each. This resulted in five times series of 1,209,600 data points each, totalling over 6 millions of data points for the 5 months. To evaluate the performance of our proposal we trained different ANN, CNN and ARIMA models for each available time series, and evaluated their ability to produce forecasts on the time series of the next month. Time series for weekdays and weekends were considered separately because we experimentally observed significant differences in their structure. In addition, we considered using a naive approach as a baseline, consisting simply in using the last observed value as prediction. For each experiment and model, mean squared (MSE) and mean absolute (MAE) errors were computed to measure the quality of predictions.

We ran a first set of experiments to compare the accuracy of the CNN, ANN and ARIMA models by computing MAE and MSE errors per month, week period (weekday or weekend) and forecasting granularity (1, 2, 4, 8, 16, 32 and 64 seconds). The obtained results show that CNN models outperform both ANN and ARIMA by an increasingly significant margin as the forecasting granularity is above the 16-second resolution. In particular, ARIMA models do not seem to obtain better results than the naive approach at any resolution. These results suggest that the analysed time series exhibit meaningful non-linear structure that can be efficiently captured by the proposed CNN models. Two additional sets of experiments were set up (1) to highlight the positive effect of including the multiresolution context (i.e. past observed values with different levels of granularity) in ANNs and in particular in CNNs, which can take further advantage of this approach through their multiple channels, and (2) to show the durability of the models, testing their ability to forecast the time series of increasingly distant future months. In this context, and given that the training procedures only take about one hour using a modern GPU, it is feasible to retrain CNN models monthly or even weekly. In addition, CNN and ANN predictions can be made in about 1e-5 seconds as opposed to ARIMA predictions, which take around 0.16 seconds. These times jointly with the observed durability of CNN models make them perfectly suitable for use in real-time scenarios and in particular to forecast the number of user sessions expected to be crossing a data center network each second.

The rest of this paper is structured as follows. Section 1 provides an overview of related research. Section 2 describes the problem setting. Section 3 presents the forecasting models we have utilized and section 4 details our proposal for improving the quality of the forecasts. Section 5 shows our experimental results and section 6 summarizes concluding remarks along with future lines of research.

## 1 Related work

The field of time series forecasting has attracted interest in many domains of application for decades. Examples of uses can be found in areas as varied as econometrics, weather forecasting and information and communication technology.

An application that has sparked significant interest in the last decades is the prediction of prices and demands in the energy market. Examples can be found in [2], where ARIMA and GARCH are combined with the wavelet transform, or [3], where a simple nearest-neighbour approach is employed. Given their ability to approximate arbitrarily complex functions, the effectiveness of neural networks as a forecasting method has been studied in different occasions. For instance, in [4], neural networks are combined with wavelets and ARIMA to predict wind speed, which can be of use in wind energy farms. The prediction of electricity prices has also been analyzed with neural networks, as in [5], where radial basis function networks are combined with ARIMA, or [6] where a special type of wavelet neural network is used. In [7], authors propose ARIMA and GARCH combined with wavelets and neural networks to predict gas prices, limiting their analysis to a time series comprised of 600 points.

Recently, a research trend called anticipatory networking has emerged. Briefly, it consists in leveraging the knowledge of the evolution of a network in order to support its optimization and improve its operation through the prediction of future network states. In particular, research works addressing link and traffic context prediction (e.g., network load, throughput, physical resource utilization) have gained momentum in the last two decades. In [8], the authors propose a combined approach using feed forward artificial neural networks, Support Vector Machines (SVMs) and dimensionality reduction techniques for path loss prediction in urban environments. In [9], a linear regression model to adjust the routing metrics in ad hoc wireless networks is presented. AutoRegressive and Moving Average (ARMA) time series filtering techniques are applied in [10] to predict near term link quality for a resource allocation algorithm for mobile networks. In the context of the prediction of Internet traffic dynamics of cellular devices, [11] proposed a combined approach using in a first stage unsupervised clustering techniques and then markov models correlating the spatio-temporal relations in order to capture the volume dynamics of aggregate Internet traffic in each cluster. With the aim of avoiding network congestion while improving the performance of applications, the authors of [12] proposed some link-level metrics suitable for application/TCP layer control jointly with an ARIMA model for their prediction. Some works propose network infrastructure optimization based on predicting the network throughput. The authors of [13] proposed ARIMA models for long term predictions to effectively forecast data rates up to 6 months in advance with a 12-hour granularity. Complementarily, in [14] and [15] shorter time scales are considered using ARIMA and GARCH. In [16], authors analyse the relation of different time scales in ARMA and FARIMA time series when applied to the prediction of network traffic dynamics. Their results show that these time series models perform better under the medium scale (minute) than under small time scales (millisecond and second), and that the performance of the FARIMA model shows no advantage over other models.

The dawn of virtualized services has enabled unprecedented flexibility in cloud and data center resource allocation. The increasing volume of Internet traffic that traverses data centers (more than 90 percent in the near term) has also made it necessary to adequately predict and shape resources in advance, in order to achieve good efficiency without compromising performance. In [17], a series of services are proposed to distribute workloads between servers. Forecasts are made by analyzing the patterns in historic traces and generating synthetic future workloads that exhibit the same dynamics. In [18], neural networks and an auto-regressive model are used to predict load demands in a cloud computing environment. In [19], the authors make use of ARIMA to predict load demands and adjust resource provisioning. The authors of [20] have recently proposed a deep convolutional neural network for detecting a phenomenon called noisy neighbour, a term used to describe a situation typical of data centers, in which several virtual machines located in the same physical machine disturb each other significantly decreasing their performance.

The soaring popularity of deep learning has spurred the development of specific methods for time series analysis and forecasting. Time sequences are commonly modeled using recurrent neural networks (RNN) [21], whose units use their own output as input at the next iteration in order to model short-term temporal relationships. A type of recurrent unit that has proved successful in different domains is the one known as long short term memory (LSTM) [22]. Deep RNNs now achieve the best results in speech recognition tasks [23], replacing the hand-crafted feature extraction methods previously employed. Another approach to time series modeling in the deep learning community is based on variations of restricted Boltzmann machines (RBM) [24], such as conditional and temporal RBMs [25]. These methods have found applications in the field of motion capture [26]. Finally, convolutional neural networks (CNN) [27] have been one of the most widely used models by the deep learning community in various domains. They are particularly useful and efficient for data with some sort of topological structure in the feature space. For that reason, they have been instrumental in achieving state-of-the-art results in computer vision [28]. However, they have also been successfully applied to sequential data like speech [29], sentences [30] and time series [31, 32]. Convolutional neural networks are particularly interesting in domains where large amounts of data need to be processed. CNNs employ sets of shared weights across the whole input, which is efficient both statistically and computationally. In addition, convolution operations are very well optimized for GPU architectures. In the domain of network traffic analysis, data are likely to be abundant and their behavior can change over time. Therefore, models should ideally be retrained periodically. To the best of our knowledge, the effectiveness of CNNs for the problem of short-term network traffic volume forecasting has not been explored. Therefore, in this paper we study the performance of this technique in this problem when compared to other methods. We describe CNNs in more detail in section 3.2.

## 2 Problem setting

We are interested in forecast short-term network traffic dynamics in a data center. Specifically, we want to forecast the number of user sessions (i.e., transport level TCP connections) expected to be crossing a network link in the range of seconds, using as input a time series signal comprised by the previous observations of this value sampled at one-second intervals.

We consider time series of the form *x*_1_, …, *x*_*n*_ where *x*_*k*_, *k* = 1, …, *n* represents the number of transport level connections active at time *k* in the analyzed network link with a granularity of one second. That is, we have one measurement every second for all the processed time periods. We consider the following generalized problem framework:

**Definition 1** *Generalized time series forecasting*

*Given a time series of the form x*_1_, …, *x*_*n*_, *two numbers* l,h∈N, *a function* $g:R^{h}\to R$, *and an error function E*, *find a function* $f:R^{l}\to R$ *such that*

$$\begin{array}{c} \sum_{i}E(f\left(x_{i_{1}},\ldots ,x_{i_{l}}\right),g\left(x_{i_{l+1}},\ldots ,x_{i_{l+h}}\right)) \end{array}$$

*is minimized*.

In its simplest incarnation, our generalized framework takes the form of standard time series forecasting: given a set of *l* consecutive entries, *x*_*i*_1__, …, *x*_*i*_*l*__, predict the value of *x*_*i*_*l*+1__. In that case, *g* is the identity and *h* = 1. However, we also consider aggregation functions of multiple future entries of the time series to evaluate the performance of our forecasting models at different levels of granularity. For instance, one might want to forecast the average value of the next 60 entries in the time series, which could be accomplished by setting *g* to be the arithmetic mean and *h* = 60. Details are given in section 4.

A successful method for this task is undoubtedly useful for cloud, data center and content service providers. For instance, it could help in detecting anomalous behavior or, if changes in demand patterns can be predicted, in adequately provisioning resources so as to optimize costs without compromising availability.

## 3 Forecasting models

There exists a wide variety of methods for time series forecasting. Among these, ARIMA is perhaps the most well-established one, due to its efficiency and simplicity of use. When the data exhibit complex nonlinearities, however, ARIMA may fail to produce a good model.

We therefore propose the use of three methods for time series forecasting. In order to examine the presence of meaningful non-linear dependencies in the data, we use artificial neural networks, due to their flexibility and ability to arbitrarily approximate continuous functions. We also employ convolutional neural networks, which can provide models for data with meaningful topology (such as time series) in an efficient manner, both statistically and computationally. We employ ARIMA as a baseline model. In this section we briefly describe these methods.

### 3.1 Artificial neural networks

Feedforward artificial neural networks (ANN) are a widely used method for pattern recognition. In essence, ANNs consist of stacked layers of linear maps followed by non-linear transformations. Layer *i* of an ANN can be represented as follows:

$$\begin{array}{c} \sigma (W_{i}x+b_{i}) \end{array}$$

where $W_{i}\in R^{m_{i}\times n_{i}}$, $x\in R^{n_{i}}$, $b_{i}\in R^{m_{i}}$, and $\sigma :R^{m_{i}}\to R^{m_{i}}$ is an entry-wise non-linear function. Here, *x* is the input to the layer. If the layer is not the first in the network, *x* is the output of the previous layer. The dimensionality of the input, *n*_*i*_, is determined either by the dimensionality of the data or by the output of the previous layer. The dimensionality of the output of each layer, *m*_*i*_, can be chosen by the user. The output of each layer is fed to the next, and the final output of the network can be specified depending on the learned task (regression, binary or multiclass classification, etc). The values of *W*_*i*_ and *b*_*i*_ are usually estimated by some variation of the gradient descent optimization algorithm.

In recent years, ANNs have enjoyed soaring popularity due to breakthroughs in understanding how to train deep networks [33], which exhibit extraordinary expressiveness and can be used for automated feature extraction [34].

### 3.2 Convolutional neural networks

Convolutional neural networks [27] are a specialized type of ANN featuring convolutional layers. Instead of the linear maps learned by ANNs, convolutional layers use convolutional filters. Convolutional filters are linear functions that are applied to the input data in a sliding-window fashion. For instance, let us denote some filter by *θ* = (*θ*_1_, …, *θ*_*w*_). Note that *θ* is a linear function mapping $R^{w}$ to R. Given an input vector $x\in R^{n}$, the *i*-th entry of the output *y* resulting from the transformation of *x* by filter *θ* is

$$\begin{array}{c} y_{i}=f\left(\sum_{k=1}^{w}\theta_{k}x_{i+k}\right) \end{array}$$

where f:R→R is a non-linear function. Filters are usually only applied where the borders of the input data permit it, resulting in a reduction of dimensionality. This reduction can be avoided using zero-padding. There exist numerous online resources for understanding convolutional networks.

The fact that the filter is applied unchanged throughout the whole feature space requires the input to have some meaningful topology, as is the case of images or time series. Convolutional networks have proved to be extremely effective for tasks such as image and speech recognition [28, 29]. Recently, the authors of this work proposed a method based on convolutional neural networks to detect noisy neighbors in cloud infrastructure which was shown to outperform other approaches [20].

In addition to their ability to exploit the topology of the input data, convolutional neural networks are efficient thanks to weight-sharing, that is, the use of the same filter weights throughout the whole input space, which helps reduce the number of parameters to be estimated.

**Fig 1:**
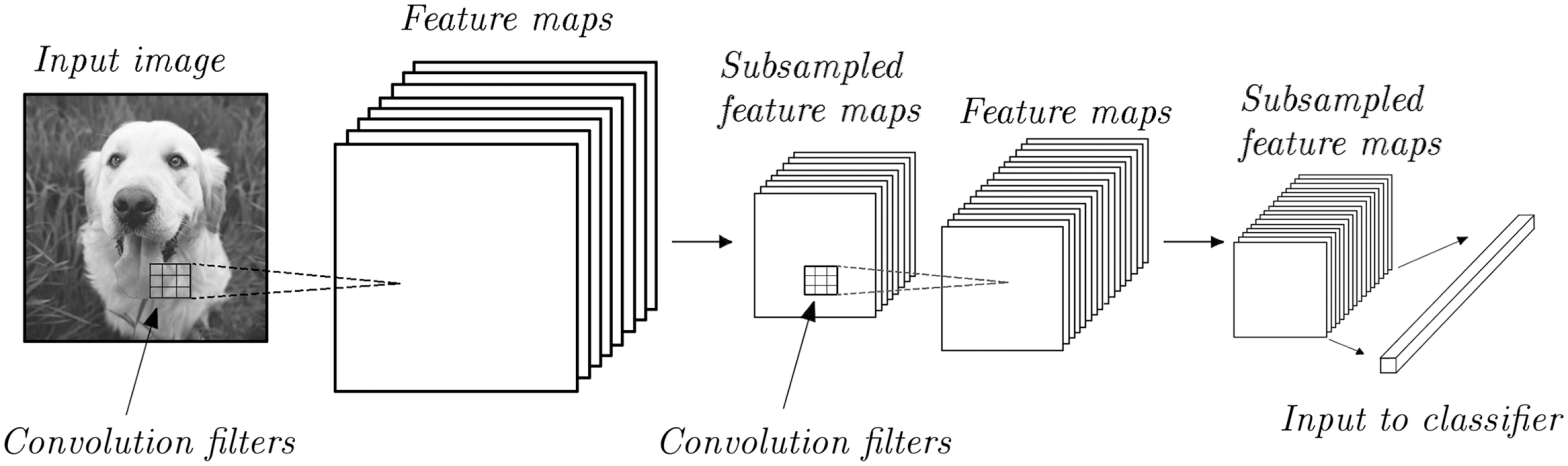
Typical convolutional network architecture for image recognition.

Fig 1 shows a typical convolutional network architecture for image recognition. The image is first processed using the convolutional filters learned by the training algorithm. This produces a set of new feature maps, or filtered versions of the image. In computer vision, filters usually learn to extract useful features such as edges in different orientations. The resulting pixels normally undergo a non-linear transformation. Another operation that is typically used in computer vision is subsampling or pooling. The rationale is that subsampled images can still convey much of the most relevant information. The number of stacked convolutional and subsampling layers can be chosen by the user. Afterwards, the final feature maps can be flattened into a high-dimensional vector that can be fed to a classifier, e.g. a fully-connected neural network. The way we use convolutional networks for time-series processing is described in section 4.3.

### 3.3 ARIMA

Autoregressive integrated moving average (ARIMA) models are a well-established method for time series analysis and forecasting. ARIMA models enjoy widespread use due to their efficiency and simplicity of use following established methodologies. Successful applications can be found in various domains, in particular in network traffic modelling and prediction [15, 35, 36].

An ARIMA(*p*, *d*, *q*) model can be characterized by the following equation:

$$\begin{array}{c} x_{t}^{\left(d\right)}=\mu +\sum_{i=1}^{p}\phi_{i}x_{t-i}^{\left(d\right)}+\epsilon_{t}+\sum_{i=1}^{q}\theta_{i}\epsilon_{t-i} \end{array}$$

where

- *p* is the number of autoregressive terms
- *d* is the number of times the series must be differenced before it becomes stationary. $x_{t}^{\left(d\right)}$ represents the value at time *t* of the time series after differencing *d* times.
- *q* is the number of lagged errors in the model
- $\phi_{i},\theta_{j}\;inR$, *i* = 1, …*p*, *j* = 1, …*q* are the coefficients to be inferred.

In our experiments we employ ARIMA as a baseline in order to determine if our approach yields any improvements when compared with traditional linear models.

## 4 Improving the quality of the forecasts

One of the main interests of network infrastructure and service providers is the possibility of making reliable forecasts of the dynamics of network traffic. In this paper we set out to determine the extent to which the short-term behavior of network traffic, namely the number of traffic flows crossing the core network of an ISP, can be predicted. In the proposed setting (see section 2), at the one-second resolution, the behaviour of traffic is often very noisy, which makes it nearly impossible to forecast the number of flows significantly better than a random guess. To improve the quality of the forecasts in this scenario, we adopt three approaches:

- An efficient method to incorporate multiresolution long-range context into the input data for the forecasting models.
- Aggregation of future values to produce more coarse-grained but more accurate forecasts.
- The use of multiple-channel convolutions to incorporate multiresolution context

We now describe these approaches in more detail.

### 4.1 Multiresolution input for modelling exponentially wide context efficiently

The most straightforward approach to forecasting is to take a vector comprising the last observations as input to the function responsible for producing a prediction. One shortcoming of this approach is that we are only taking into account the most immediate events, but ignoring the context of long-term dynamics. To increase the amount of contextual information one can simply increase the size of the sample used to predict, i.e., instead of 60 observations we can take 120. Unfortunately, this approach doubles the dimensionality of the input data, which can have significant impacts on computational and statistical efficiency, providing only context for a period twice as long as the previous one.

To tackle this problem we propose the following approach. We work under the hypothesis that as we look further back into the past, the most relevant elements of the information become more coarse-grained. Therefore, we can aggregate data from the distant past to produce lower resolution time series segments and, presumably, obtain almost as much information as we would by taking all observations into account. More precisely, to predict the value *x*_*t*_, we enrich data instances with sets of values in the following form:

$$\begin{array}{c} \left\{\gamma \left(\left[x_{t-1-\left(i+1\right)*2^{c}},x_{t-1-i*2^{c}}\right]\right):i=1,\ldots ,w\right\} \end{array}$$

where

- γ:I→R (where *I* is the set of intervals of consecutive observations) is an aggregation function, which maps intervals of the form [*x*_*a*_, *x*_*b*_] for some *a* ≤ *b* to an aggregation of the values contained therein.
- *w* is the size of the input window, that is, the number of observations that we use as input at each level of aggregation.
- *c* is the exponential context degree, which we define as the base-2 logarithm of the width of the interval aggregated by *f*.

This way we can incorporate exponentially wide time windows into the input of our predictors using a linear amount of data. As an example, consider we want to take a window of length 60 and enrich it with exponential context degrees of 1, 2 and 3. Then, a single data instance of our input data will be comprised of the following entries.

$$\begin{array}{c} x_{t-60},\ldots ,x_{t-3},x_{t-2},x_{t-1}\\ \gamma \left(\left[x_{t-120},x_{t-119}\right]\right),\ldots ,\gamma \left(\left[x_{t-4},x_{t-3}\right]\right),\gamma \left(\left[x_{t-2},x_{t-1}\right]\right)\\ \gamma \left(\left[x_{t-240},x_{t-237}\right]\right),\ldots ,\gamma \left(\left[x_{t-8},x_{t-5}\right]\right),\gamma \left(\left[x_{t-4},x_{t-1}\right]\right)\\ \gamma \left(\left[x_{t-480},x_{t-473}\right]\right),\ldots ,\gamma \left(\left[x_{t-16},x_{t-9}\right]\right),\gamma \left(\left[x_{t-8},x_{t-1}\right]\right) \end{array}$$

That is, with 240 data we are modeling the events up to 480 seconds in the past, which would require 480 data if we used all elements of the time series. This saving is significant if we take into account the effects of the curse of dimensionality, that is, the need for an exponentially greater amount of data to learn a function as the dimensionality of the input increases linearly.

### 4.2 Coarse-grained long-term forecasts

Our preliminary observations show that complex methods for time series forecasting at the one-second resolution in the domain described in section 2 do not outperform naive approaches (i.e., simply forecasting the last observation) by a wide margin. The reason for this is most likely the amount of noise present in the signal sampled at the one-second frequency. Our intuition is that part of this noise could be explained via exogenous variables related to the virtual and physical machines running on the data center (e.g. CPU and memory usage, or inbound and outbound network packets of the corresponding virtual machines). As a future extension of this work, it would be interesting to analyze whether a richer time series with this information could help to improve the quality of fine-grained forecasts.

To deal with this problem we propose to explore the predictability of our data at wider time ranges, by trying to forecast the mean values over a time period. The prospects of this approach are supported by both intuition and theory. Intuitively, the behavior of network traffic is expected to be more structured in the long term, where randomness plays a lesser part in the dynamics. Theoretically, the scale of aggregated observations increases linearly in the number of components, while the standard deviation of a sum of independent Gaussian random variables increases as a fractional power.

Formally, if *ϵ*_*i*_, *i* = 1, …, *n* are *n* i.i.d. Gaussian variables such that $\epsilon_{i}∼N\left(\mu ,\sigma^{2}\right)$ for all *i*, then

$$\begin{array}{c} \sum_{i}\epsilon_{i}∼N\left(n\mu ,n\sigma^{2}\right) \end{array}$$

A single scaled Gaussian variable $\frac{\epsilon_{i}}{n}$, however, is distributed as

$$\begin{array}{c} \frac{\epsilon_{i}}{n}∼N\left(\frac{\mu }{n},\frac{\sigma^{2}}{n^{2}}\right) \end{array}$$

Therefore,

$$\begin{array}{c} \frac{1}{n}\sum_{i}\epsilon_{i}∼N\left(\mu ,\frac{\sigma^{2}}{n}\right) \end{array}$$

If we assume that our time series has a noise component of i.i.d. Gaussian variables and we take the mean of *n* separate values, the standard deviation of the noise of *n* aggregated values will thus be $\frac{\sigma }{\sqrt{n}}$. That is, the standard deviation of the noise will be attenuated as we aggregate values, or equivalently, increase the time scale of the predictions.

To take advantage of this result, we train our models not only to predict the exact value of the time series one or more seconds ahead, but also to predict the average value 2, 4, 8, 16, 32 and 64 seconds in advance. This of course results in more coarse-grained forecasts and therefore larger absolute errors, but as we show in our experiments, the error with respect to the variability of the data is drastically decreased. We limit our aggregation to the one-minute time scale, as forecasts in this range might be very valuable for certain applications that arise in data center contexts, such as real-time resource provisioning optimization [1].

### 4.3 Multiple-channel convolutions for incorporating context

In order to take further advantage of the multiresolution context added as explained in section 4.1, we feed each of the aggregated series into a different channel of the first layer of our convolutional network models. Convolutional networks learn a separate filter for each input channel. The output after processing the input with these separate filters is added together. This way, the relationship between the different channels is modeled by the network.

The way we employ convolutional networks is this: a set of convolutional filters, whose number and width are manually chosen, are learned for each sub-time series (see section 4.1), which are regarded as separate input channels. After traversing all convolutional layers (whose number is also manually chosen), the input undergoes the transformations learned by a fully connected neural network. The resulting forecast is the value of a single, linear output unit.

The first convolutional layer uses a set of three-channel (exponential context degrees of 0, 1 and 2) convolution filters of size *c*. We zero-pad the input data to preserve its dimensionality. The resulting vectors are further processed by similar convolutional layers, with as many channels as convolution filters in the previous layer. We can therefore define the entries output by filter *f* of convolutional layer *l* at position *i* given an input record *x* as

$$\begin{array}{c} a_{f,i}^{\left(l\right)}=\{\begin{array}{cc} \rho (\sum_{j=1}^{3}\sum_{k=1}^{c}\theta_{fjk}^{\left(l\right)}x_{j,i+k-\lfloor c/2\rfloor }+b_{fl}) & \text{if}\;l=1\\ \rho (\sum_{j=1}^{n(l-1)}\sum_{k=1}^{c}\theta_{fjk}^{\left(l\right)}a_{j,i+k-\lfloor c/2\rfloor }^{\left(l-1\right)}+b_{fl}) & \text{otherwise} \end{array} \end{array}$$

- *x*_*j*, *i*_ is the value of channel *j* at position *i* of the input record (if *i* is non-positive or greater than the input window, then *x*_*j*, *i*_ = 0).
- $\theta_{fjk}^{\left(l\right)}$ is the value of channel *j* of convolution filter *f* of layer *l* at position *k*, and *b*_*fl*_ is the bias of filter *f* at layer *l*.
- *c* is the width of the convolution filters
- *n*(*l*) is the number of convolution filters at layer *l*.
- *ρ* is the non-linear activation function.

**Fig 2:**
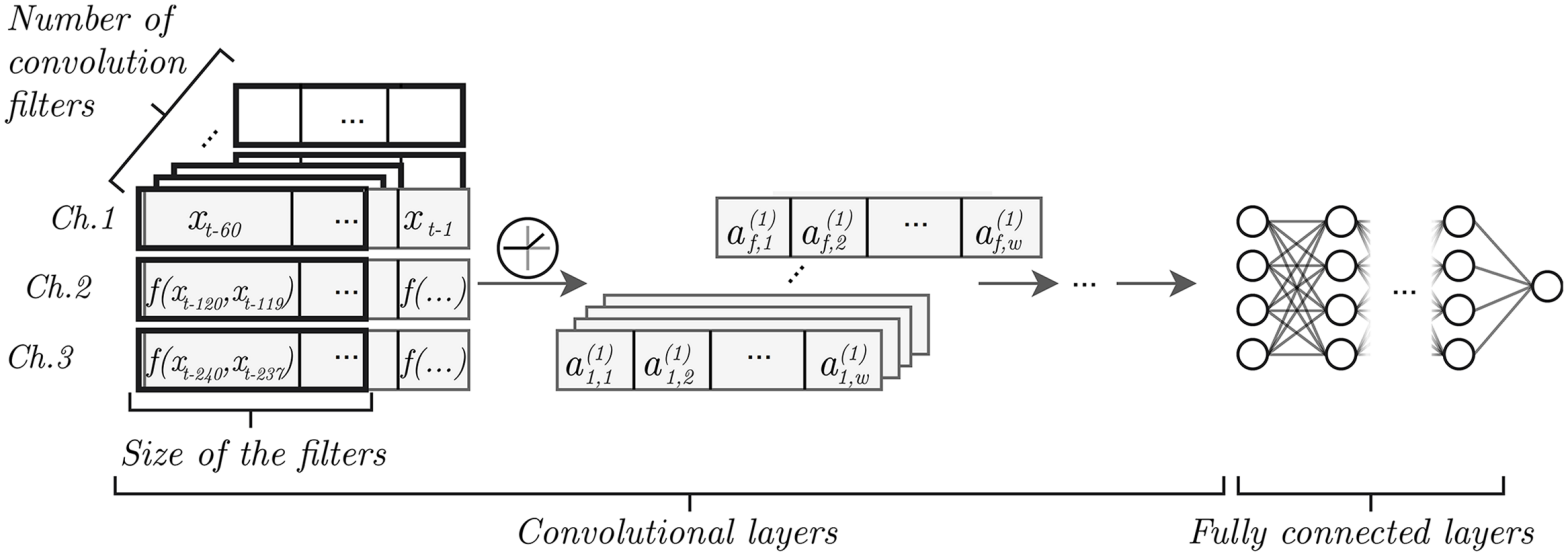
Our convolutional network architecture for time series forecasting.

Fig 2 shows a depiction of our convolutional network architecture for time series forecasting. Each of the input sub-time series will be processed by a multiple-channel convolution filter, each of which will produce a new time series. The resulting set of time series can then be processed by a new convolutional layer as a new multiple-channel time series. In this paper, however, we limit the network to one convolutional layer to determine whether this is enough to outperform other models.

## 5 Experiments

In order to validate the performance of our proposed models we carried out a series of experiments on a real traffic data set described in section 5.1. Our main goals were the following:

- To determine whether the multiresolution context helps improve the quality of the forecasts.
- To determine whether our approach based on convolutional networks (CNNs) offers any advantage over ARIMA and feed forward fully connected neural networks (ANNs).
- To determine the time scale at which forecasts can be made with good results.

Our experiments were also intended to shed light on one of the main questions posed in this paper: can neural networks, and convolutional networks in particular, provide a robust forecasting model in a complex real-time scenario where traditional methods like ARIMA would require significant preprocessing and analysis?

In the rest of this section we describe the employed data set, the approaches taken to run the experiments, and finally we discuss the obtained results.

### 5.1 Data

The data used in our experiments is a subset of the ONTS data set, a network traffic trace collected at the core network of a medium-sized Spanish Internet Service Provider over a period of three years in the context of the FP7-ONTIC research project (http://ict-ontic.eu). This network processes an average of 200,000 packets per second. ONTIC project was funded by the Seventh Framework Programme of the European Commission and access to the ONTS data set can be requested at http://ict-ontic.eu/index.php/onts-data/onts-request-access.

**Fig 3:**
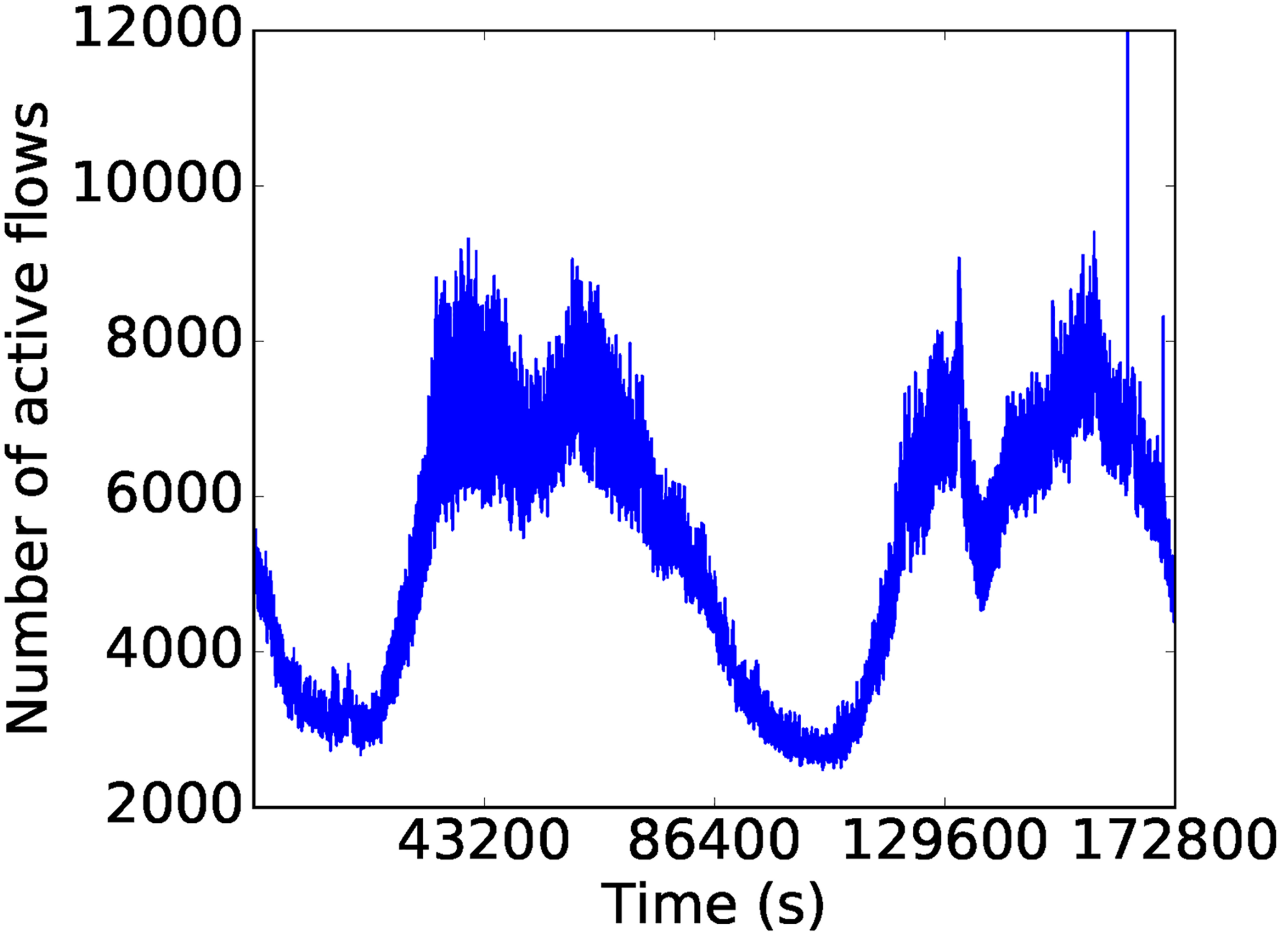
The time series from a weekend in February.

**Fig 4:**
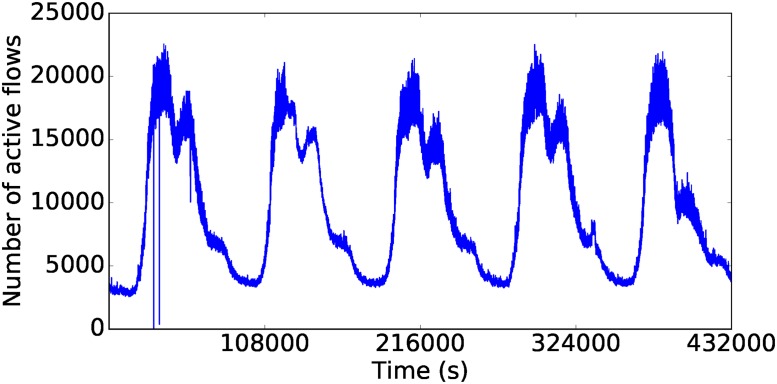
The time series from five weekdays in March.

The collected raw data set consists of network packets in PCAP format (https://en.wikipedia.org/wiki/Pcap). We processed the data in order to transform them into a time series representing the number of active TCP (Transport Control Protocol) flows at each second. This resulted in a time series of 86,400 values per day. To accomplish this, the raw packets were aggregated into 5-tuple flows (that is, groups of packets sharing source and destination IP address and ports, as well as transport protocol) using Tstat tool version 3.0 (http://tstat.polito.it/). We only kept TCP flows as they constituted the dominant protocol. The resulting records were processed to count the number of flows active at each second. This part of the process was done in parallel using Apache Spark, a distributed computing platform based on Resilient Distributed Datasets [37]. The time required to process the data corresponding to one month was three days. In total we analyzed 5 months of traffic data, out of which we extracted 75 days (2 weeks per month) to generate the data for our experiments. The whole preprocessing process therefore took over two weeks to complete and resulted in a time series representation of approximately 1,300 million flows, at an average of 18.7 million flows per day. Figs 3 and 4 exemplify a plot of the time series corresponding to a weekend from the month of February and a working week from the month of March. The long term regularities are evident, save for a few peaks or valleys probably due to attacks or service downtime.

In order to obtain a representative sample of the behaviour of the traffic, we employed data from February 2016 to July 2016 and for each month we used two weeks of data chosen at random among the available. We omitted the month of May due to a significant gap caused by a temporary breakdown of the traffic collection system.

To evaluate the performance of our ANN and CNN models we trained them on data from one time period (i.e., one month) and tested their ability to make predictions on data from a different one. In the case of ARIMA, in order to make a forecast of a time series at time *t*, we simply fit the model on past values of the series and had it forecast one value. The approaches taken to model training and fitting are explained in more detail in section 5.2. We partitioned our data set into various partitions, named *P*1, *P*2, ‥*P*10, in order to evaluate our models on different periods of time. The results training-test pairs are listed in Table 1. Given the significant differences in the behavior of network traffic during weekdays and weekends, we treated these two cases separately.

**Table 1:** Training-test pairs used to train neural networks.

Partition	Training set	Test set
P1	Weekend February	Weekend March
P2	Weekend March	Weekend April
P3	Weekend April	Weekend June
P4	Weekend June	Weekend July
P5	Weekend July	Weekend February
P6	Weekdays February	Weekdays March
P7	Weekdays March	Weekdays April
P8	Weekdays April	Weekdays June
P9	Weekdays June	Weekdays July
P10	Weekdays July	Weekdays February

In addition to the raw time series data, we also tested the abilities of the models to make forecasts at different time scales by aggregating data as explained in section 4.2. Specifically, we transformed the original time series to generate six new time series, in which each entry represents the mean of 2, 4, 8, 16, 32 and 64 steps ahead respectively. The tasks therefore amount to a total of 70 (7 time series for each training-test pair).

Finally, we analyzed the intrinsic variability of the data to determine the extent to which our forecasts are better than simply random guessing. Tables 2 and 3 show the variance and the mean absolute deviation respectively of the 1st-differenced time series for each period. These values can be compared to the mean squared error (MSE) and mean absolute error (MAE) attained by our models. Each month should be compared to the results obtained using it as test set. For instance, the MAE of partition P1 should be compared to the mean absolute deviation of March weekends, and the MSE of partition P2 should be compared to the variance of April weekends

**Table 2:** Variance of the time series after first difference for each month.

Month	1	2	4	8	16	32	64
Weekends March	3591.60	6606.43	14937.72	31830.12	52047.85	66718.89	68507.82
Weekends April	2655.34	3873.81	7999.51	16098.53	26722.44	33981.23	36699.06
Weekends June	1733.05	2862.25	5688.17	11898.44	21269.30	28821.85	33730.99
Weekends July	1643.49	2531.09	4734.81	9714.07	17106.02	22215.90	25453.01
Weekends February	3211.48	5447.91	12067.02	28510.32	55535.63	76269.86	82409.18
Weekdays March	12843.68	29425.97	73799.15	159208.98	256177.69	273978.96	242211.62
Weekdays April	9302.60	19196.10	40268.44	72689.55	104464.70	107800.58	110882.34
Weekdays June	9820.81	16200.19	34540.22	62475.00	92136.53	101665.32	102451.20
Weekdays July	9223.68	16391.68	34210.20	59340.96	87267.06	99300.61	100134.27
Weekdays February	14566.07	31719.60	75482.06	148872.50	230102.49	253723.76	235419.03

**Table 3:** Mean absolute deviation of the time series after first difference for each month.

Month	1	2	4	8	16	32	64
Weekends March	37.77	49.61	74.00	109.65	141.68	166.80	187.46
Weekends April	29.21	37.60	55.42	80.40	104.00	122.16	138.38
Weekends June	27.26	33.68	45.63	64.79	89.26	113.14	135.00
Weekends July	27.53	33.57	44.79	62.58	84.60	101.51	113.29
Weekends February	33.57	41.41	58.52	88.85	131.59	172.03	201.93
Weekdays March	48.98	67.69	105.07	166.29	242.79	302.19	335.65
Weekdays April	36.36	52.41	80.65	122.51	165.78	199.52	227.96
Weekdays June	41.68	54.19	77.98	113.34	154.60	188.89	217.43
Weekdays July	45.46	55.00	78.08	113.53	156.44	192.19	218.99
Weekdays February	50.49	68.44	104.05	161.17	233.19	293.99	337.44

### 5.2 Model fitting

In this subsection we explain how each of the models was fitted and evaluated.

#### 5.2.1 Neural networks

The approach for training and evaluation was the same for both fully connected (ANNs) and convolutional neural networks (CNNs). We trained two different ANNs and two different CNNs for each time period, one with and one without multiresolution input (see section 4.1). The networks without multiresolution input simply take the previous time series values (60 and 240 in two different experiments) as input to produce a forecast. On the other hand, the input to multiresolution networks incorporated past data with exponential context degrees of 0, 1 and 2. This resulted in a 180-dimensional vector representing data from up to 240 seconds in the past. While the reduction in dimensionality from 240 to 180 might seem small, it should be noted that the amount of data needed to learn a function can increase exponentially in the number of dimensions. Therefore, every reduction is valuable. In the case of ANNs, the input was flattened into a 180-dimensional vector (60 values for each degree), while in the case of CNNs, each of the 3 resulting sub-time series was fed into a different channel (see section 4.3 for details).

We trained the networks to minimize the mean squared error between their predictions and the true future observations. We used different models to predict future time steps at different levels of aggregation (always aggregating by taking the mean), specifically at 1, 2, 4, 8, 16, 32 and 64. In our generalized time series forecasting framework (see section 2), this would correspond to the function *g* representing the arithmetic mean and *h* taking the values from 1 up to 64.

A separate model was trained for each of the above described approaches for each time period, resulting in 140 ANN and 140 CNN models in total (2 ANNs and 2 CNNs per partition). In each case, a random subset containing 10% of the data was kept for validation, unused for training. The training process went on until no improvement on the validation split was observed for 50 epochs. Afterwards, the weights that yielded the best validation error were kept. We tuned the hyperparameters of the networks via random search. We monitored the error on the validation set in order to choose the hyperparameters. Afterwards, we employed these hyperparameters to train the network (again monitoring validation error to decide when to stop) and evaluated the error obtained on the test set, consisting of data from an different period of time. In the end, we chose a set of hyperparameters that exhibited small errors on the validation set but that allowed us train the networks in a reasonable time (around 1 hour approximately). The resulting hyperparameters are specified below separately for ANNs and CNNs. The employed training algorithm was Adam [38].

The networks were designed and trained using the keras (https://keras.io/ library with the Theano deep learning framework as backend. The training was done on an Asus ROG Strix Geforce GTX 1080 8GB GDDR5X GPU equipped with 2500 CUDA cores and 8GB of RAM. The whole training process took around 10 days.

**Feed-forward fully connected neural networks (ANNs)** We used a network of one intermediate layer of 60 units with ReLU activation, and a final single linear unit for the prediction. The ReLU activation function can be defined as

$$\begin{array}{c} \rho :R\to R^{+} \end{array}$$ (1)

$$\begin{array}{cc} & x\mapsto \max\{0,x\} \end{array}$$ (2)

All weights were slightly regularized using *l*2-norm penalty terms of *λ* = 3.619*e* − 08. Dropout, a regularization method which is usually very effective for classification, was problematic for this task, so it was not used.

It should be noted that we ran extensive tests with larger networks, adding more layers and units, but we did not observe noticeable improvements. We therefore employed this configuration, which exhibited good performance while maintaining training times within reasonable limits.

**Convolutional neural networks (CNNs)** We used a single convolutional layer with 30 filters of width 15. We used zero-padding, so the transformed time series did not lose any dimensionality. The weights were regularized with an *l*2-norm penalty of *λ* = 2.477*e* − 08.

The output of the convolutional layer was flattened into a high dimensional vector, then processed by a single fully connected layer like the one used for the artificial neural network model, with 60 units and an *l*2-norm penalty of *λ* = 3.619*e* − 08.

For the tasks of predicting 32 aggregated steps ahead, we noticed that in some cases the CNN models presented large MSE values, while the MAE remained at acceptable levels. This is likely due to the presence of rare events either in the training or in the test data. The higher complexity of the CNN models makes them more sensitive to overfitting, causing them to potentially produce large errors given certain inputs. Even if those flawed out puts are rare, their large magnitude can have a dramatic impact on the MSE, which accentuates errors quadratically. To prevent this from happening, we increased the regularization penalty in the CNN models for predicting 32 aggregated steps ahead, and set it to *λ* = 2.477*e* − 06 at convolutional layers and *λ* = 3.619*e* − 06 at fully-connected layers.

#### 5.2.2 ARIMA

The ARIMA(*p*, *d*, *q*) model was fit in a different fashion from what was done with ANNs and CNNs. The reason is that fitting an ARIMA model is much more efficient and, as opposed to neural networks, it can benefit from the availability of new data in real time. An AR model (which is what we used in our experiments) can be fitted to a series of thousands of entries and produce a forecast in under a second on moderately powerful hardware, while updating (i.e., re-training) an ANN or CNN model with a newly arrived batch of thousands of instances for producing a real-time forecast is infeasible.

We followed the Box-Jenkins methodology to fit linear time series models [39]. To obtain a prediction by ARIMA at time *t*, we fit the model to the previous *k* values, that is, to the sub-time series comprised of the values in the time interval [*x*_*t* − *k*_, *x*_*t* − 1_]. We determined experimentally that *k* = 4,000 produced good results. In order to get an estimate of how this approach is expected to perform, for each time period (e.g. weekends in February) we took 30,000 such sub-time series sampled at random without replacement, and measured the error of the 30,000 resulting forecasts with respect to the corresponding actual values. This way we could obtain an accurate estimate of the expected error while keeping the experiments manageable on our hardware.

**Fig 5:**
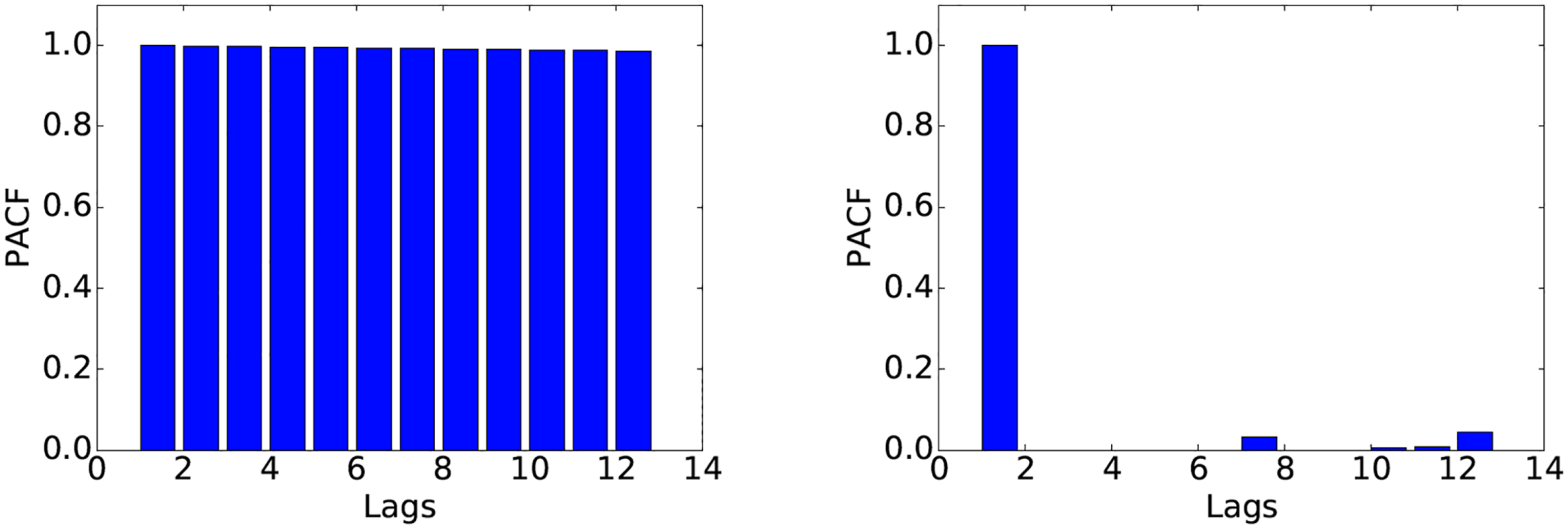
ACF (left) and PACF (right) of the raw data.

**Fig 6:**
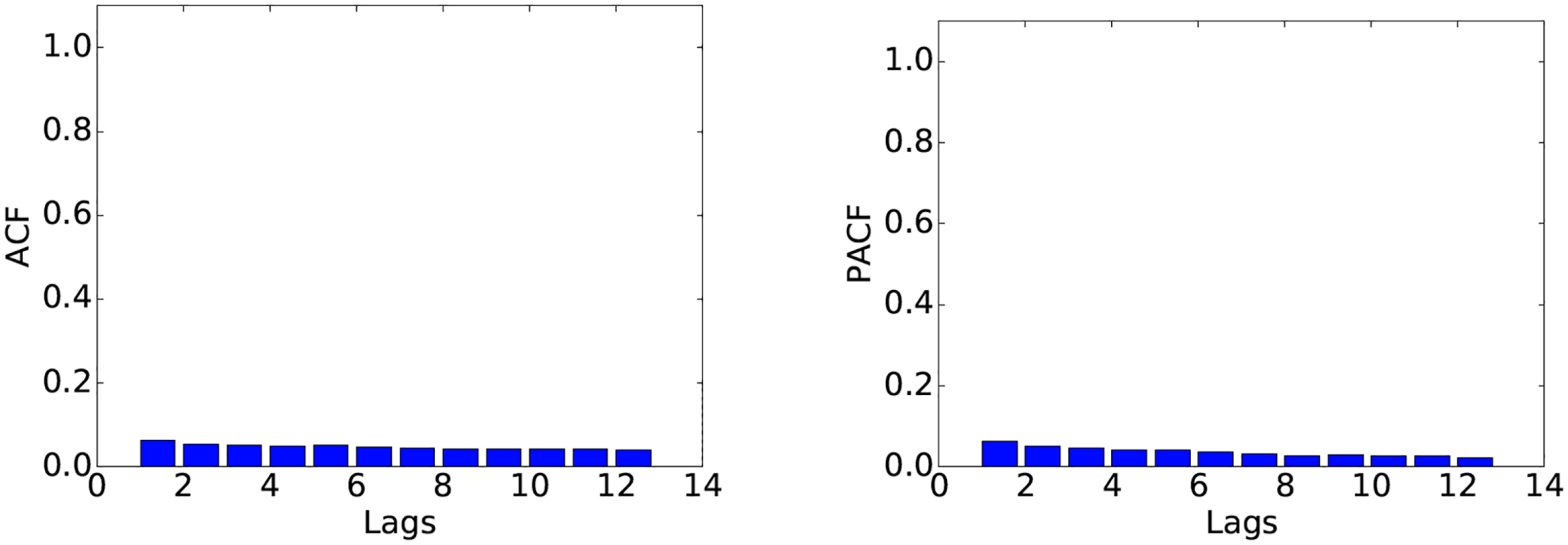
ACF (left) and PACF (right) after first difference.

We determined the values of *p*, *d* and *q* based on the data corresponding to weekends in February. We first choose the value of *d*, i.e., the degree of differencing. The raw data (Figs 3 and 4) shows clear non-stationarity, an insight that is reinforced by the autocorrelation function plot (ACF) in Fig 5. After applying one degree of differencing, the series becomes stationary, and the autocorrelation becomes small at all steps, as shown in Fig 6. We therefore set *d* = 1. Neither the autocorrelation (ACF) nor the partial autocorrelation (PACF) functions (Fig 6) exhibit significant peaks, which is consistent with the intuition that at the one-second scale, the series is highly noisy. Therefore, no significant improvements can be expected from using a model other than ARIMA(0, 1, 0). Nevertheless, since the PACF plot shows a noticeable difference between the first and second lags, we also fit an ARIMA(1, 1, 0) model to check whether it would provide any performance improvement. Preliminary experiments with other choices of *p*, *d*, *q* did not show clear advantages.

**Fig 7:**
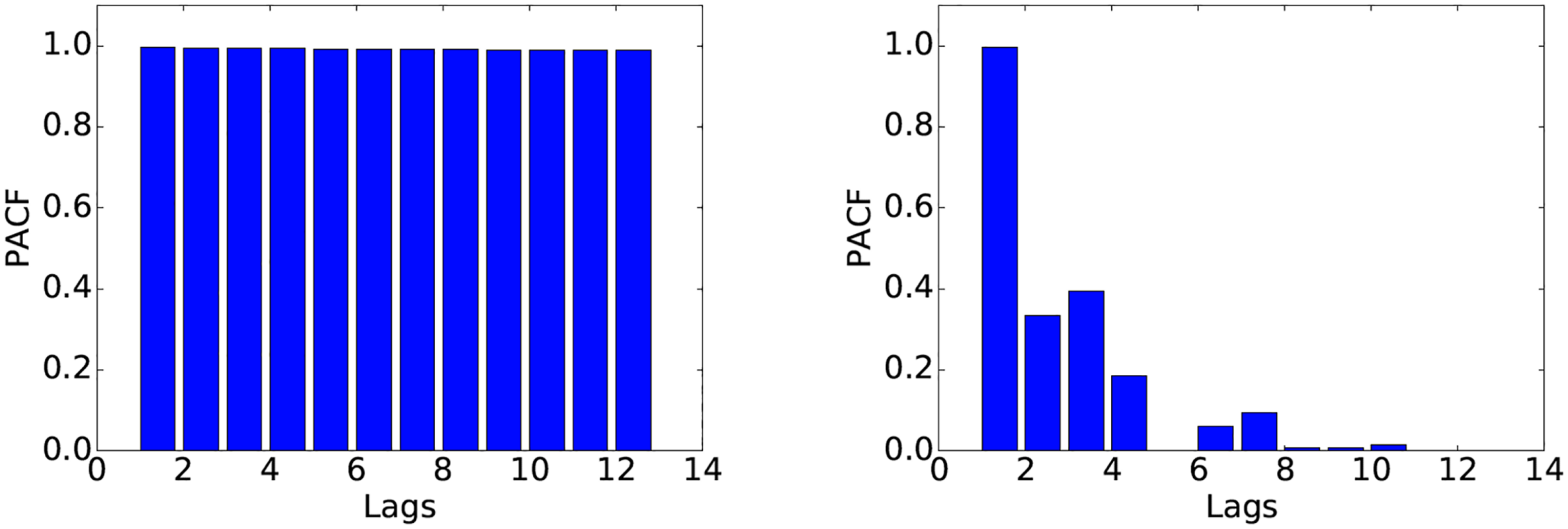
ACF (left) and PACF (right) of the raw data at the 64-second resolution.

**Fig 8:**
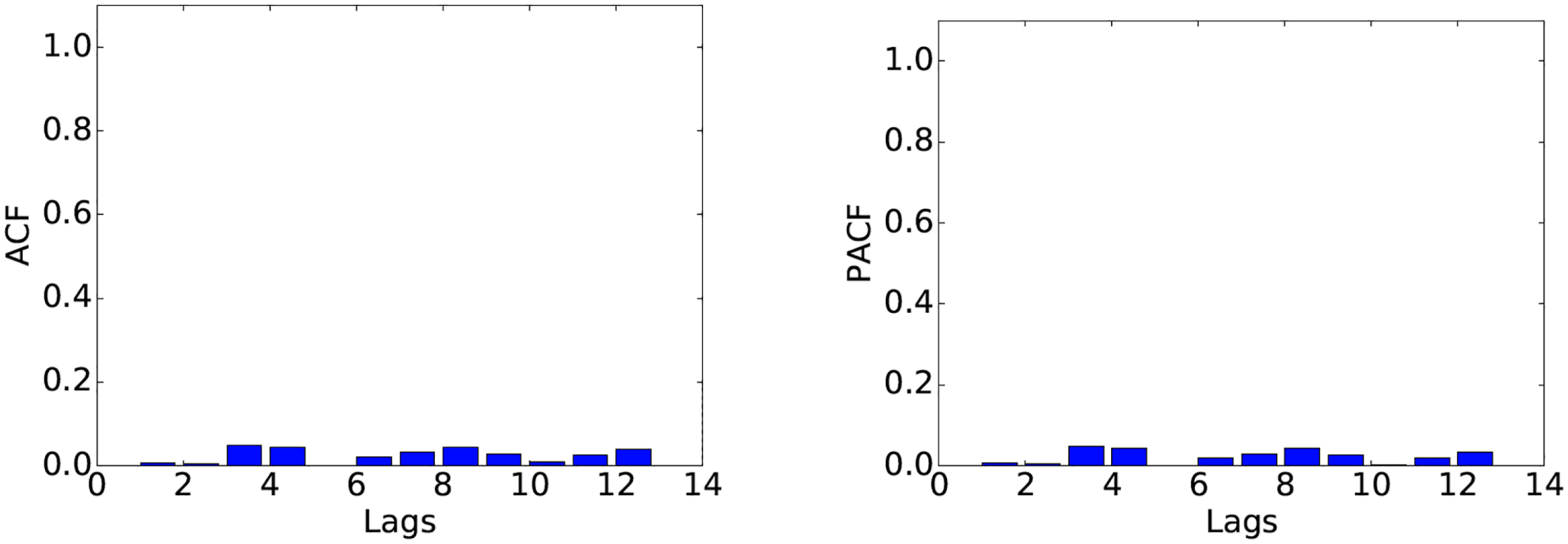
ACF (left) and PACF (right) after first difference at the 64-second resolution.

In order to determine whether ARIMA is suitable for these data at a higher time scale, we examine the ACF and PACF of the series at the 64-second resolution. These plots (Fig 7) reveal a similar behaviour to the one exhibited at the 1-second scale. After differencing, the series shows no significant autocorrelation (Fig 8). This observation is crucial, as it suggests that ARIMA is not a viable model for these data even at the 64-second scale.

In order to predict an aggregation of *n* future values, as explained in section 4.2, we considered two approaches.

1. In the first approach, we simply aggregated the input series at the corresponding time scale by taking the mean value every 2, 4, 8, 16, 32 or 64 entries, and then fitted an ARIMA model to the aggregated series.
2. In the second approach, we fitted the ARIMA models to the time series at the one-second time scale, and had them produce *n* forecasts. We then took the mean of the produced forecasts and measured its error with respect to the mean of the true *n* future values in the time series.

None of the two approaches showed clear superiority over the other, so we adopted approach 2 as it exhibited slightly higher stability.

### 5.3 Results

In addition to ARIMA, ANNs and CNNs, we evaluated a naive approach, which consisted simply in using the last observed value as a prediction. This allowed us to evaluate how much of an improvement our methods yield with respect to a straightforward, nearly zero-cost approach.

We measured both the mean squared error (MSE) and the mean absolute error (MAE) of the predictions output by the different models. The metrics are defined as follows. Given a time series (*x*_1_, …, *x*_*n*_) and a series of forecasts $\left({\hat{x}}_{1},\ldots ,{\hat{x}}_{n}\right)$,

$$\begin{array}{c} MSE=\frac{1}{n}\sum_{i=1}^{n}{\left(x_{i}-{\hat{x}}_{i}\right)}^{2} \end{array}$$

$$\begin{array}{c} MAE=\frac{1}{n}\sum_{i=1}^{n}\left|x_{i}-{\hat{x}}_{i}\right| \end{array}$$

**Fig 9:**
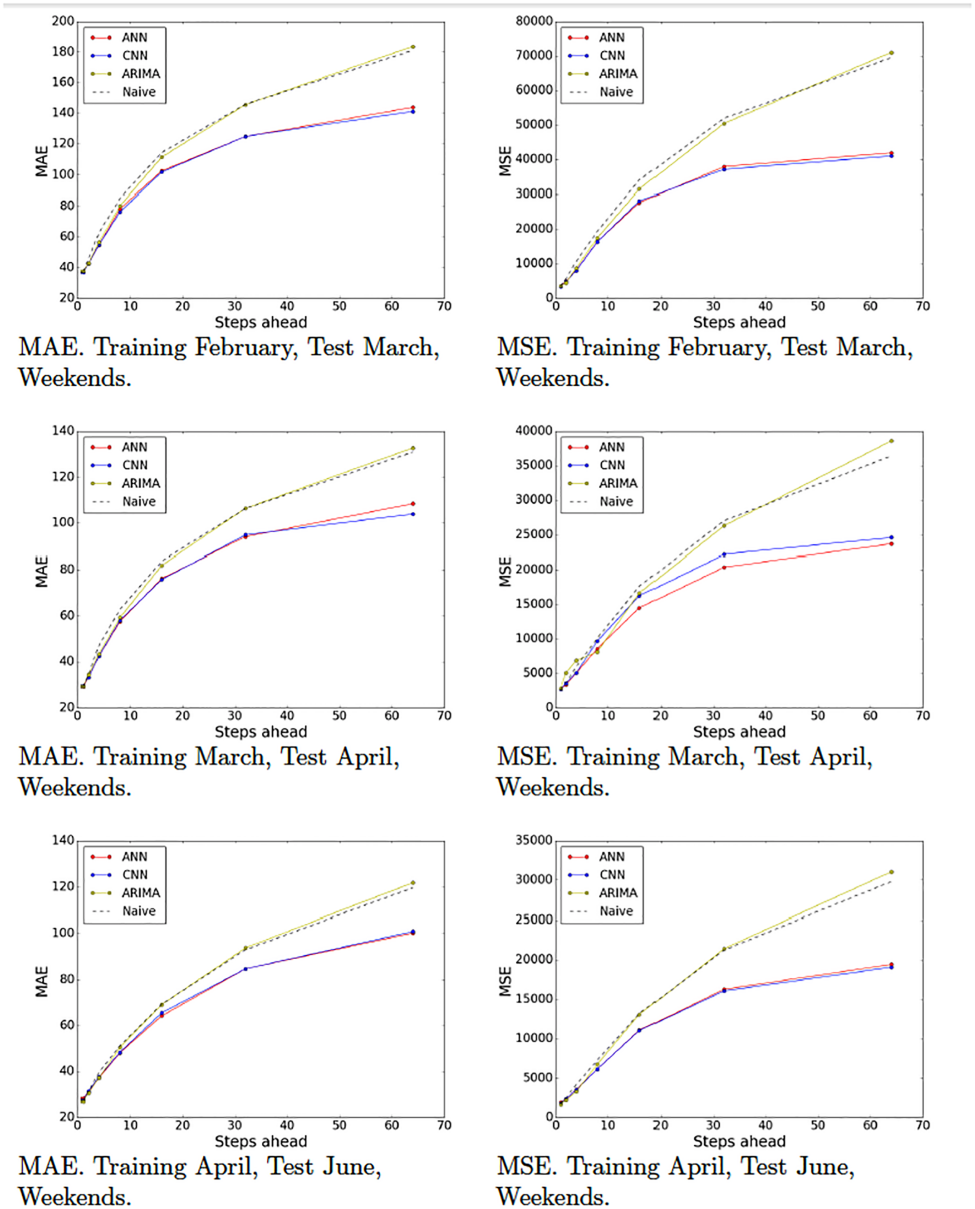
Model tests (February, March and April). Naive, ARIMA and ANN and CNN with multiresolution context. Weekends.

**Fig 10:**
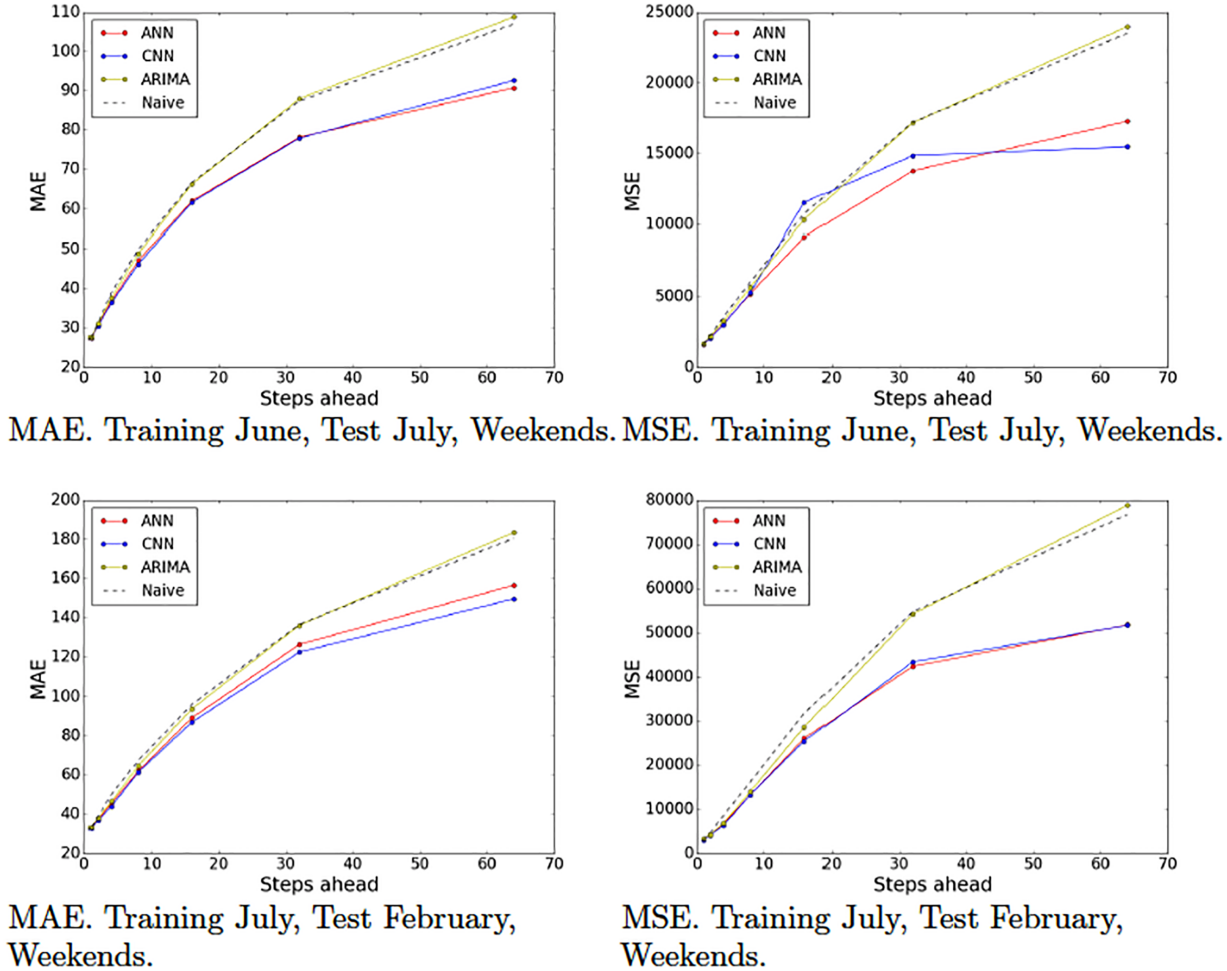
Model tests (June and July). Naive, ARIMA and ANN and CNN with multiresolution context. Weekends.

**Fig 11:**
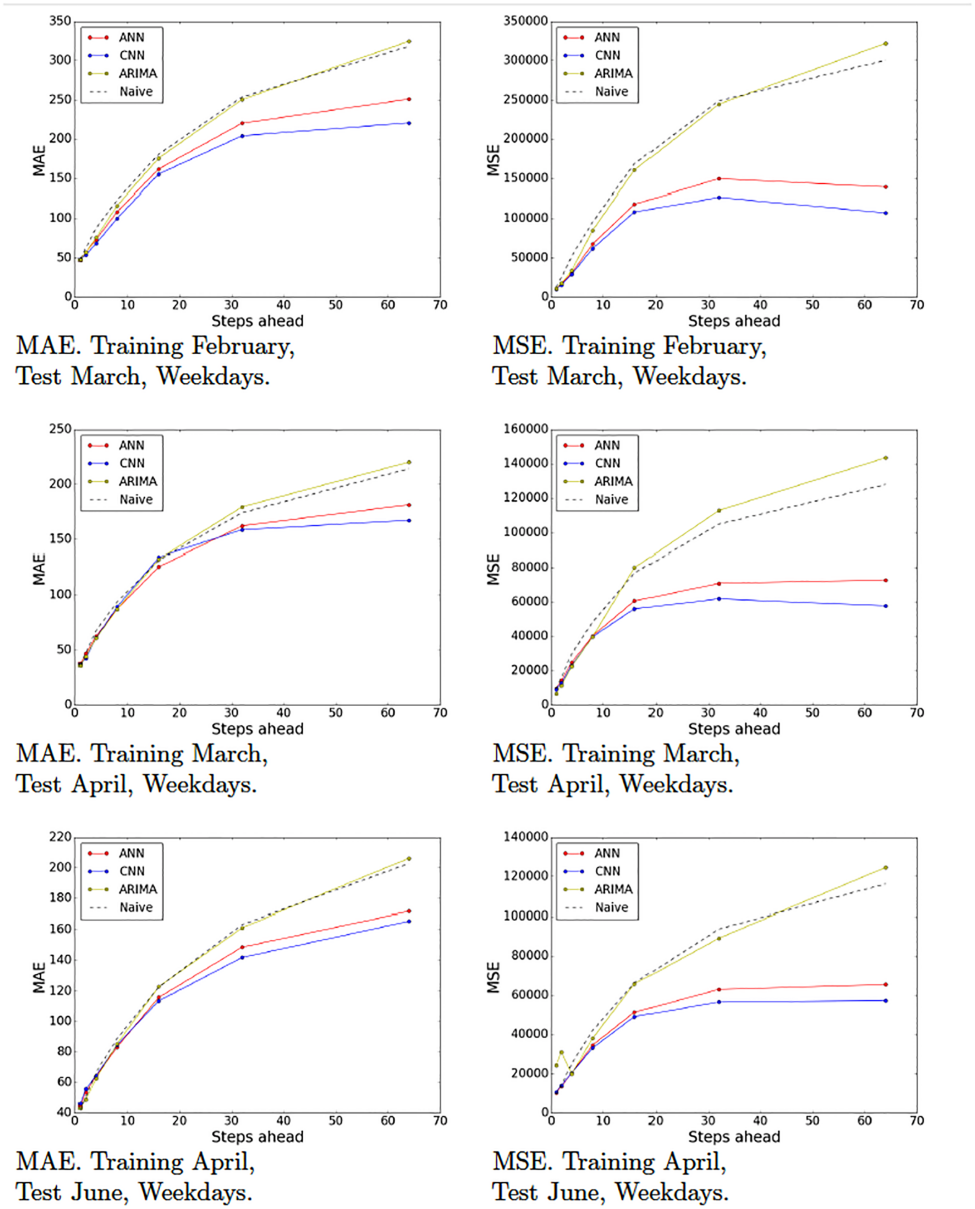
Model tests (February, March and April). Naive, ARIMA and ANN and CNN with multiresolution context. Weekdays.

**Fig 12:**
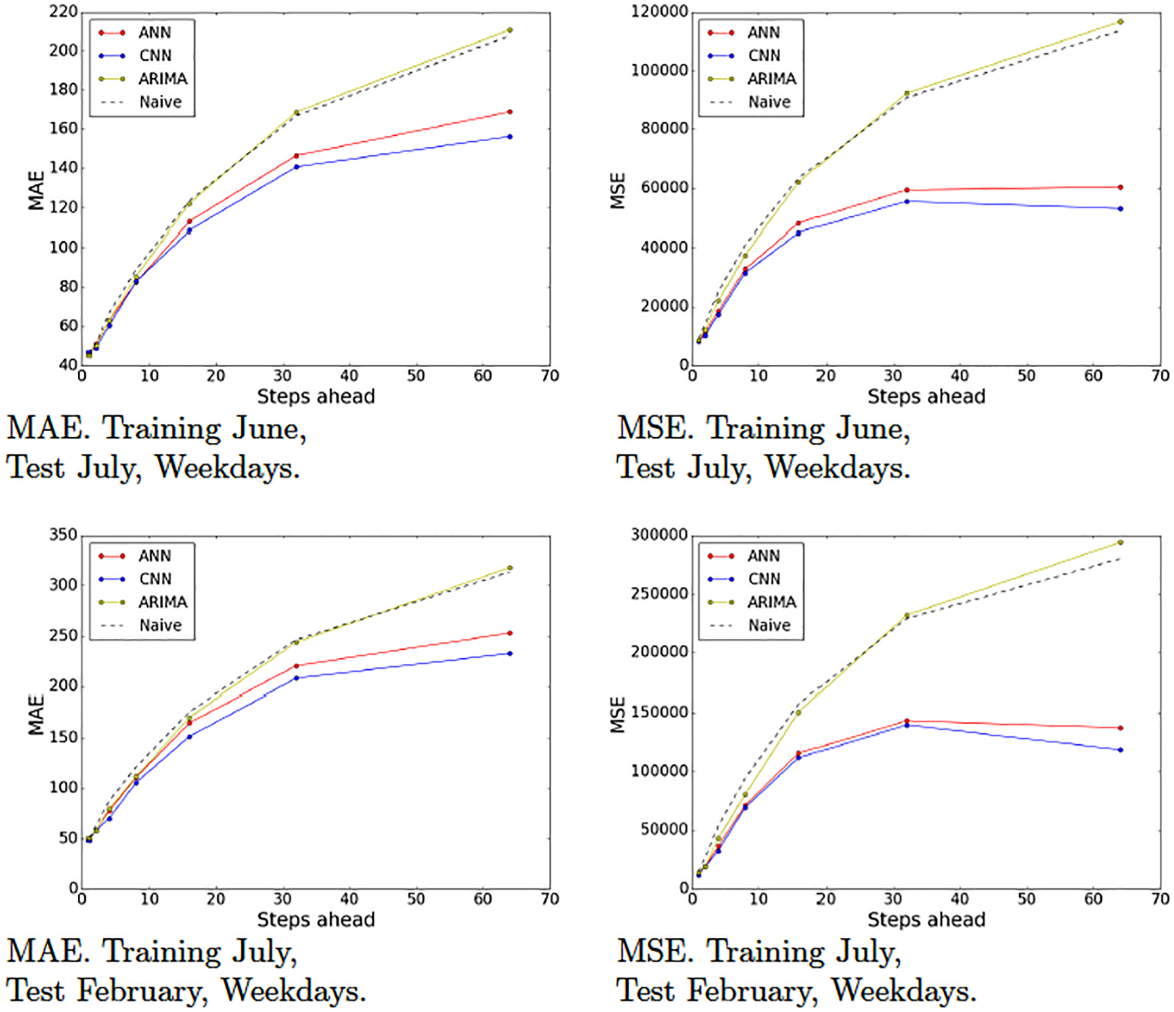
Model tests (June and July). Naive, ARIMA and ANN and CNN with multiresolution context. Weekdays.

Recall that we partitioned our data set into various partitions in order to evaluate our models on different periods of time and generated 10 training-test pairs (Table 1). Figs 9–12 show the MAE and the MSE for the three models (ARIMA, ANNs with multiresolution context and CNNs with multiresolution context) and the naive approach on each training-test pair.

These results provide interesting insights. First of all, we can see that at very short-term forecasts ANNs and CNNs are approximately on par with ARIMA and the naive approach. This suggests, as we previously hypothesized, that the amount of noise at this time scale is too high, which would render the attempts to make reliable forecasts futile. Second, at wider time scales, the improvement yielded by ANNs and CNNs is much more significant, suggesting that anticipatory decisions could be taken in our data center around the 30-seconds scale. Third, even though, contrary to our expectations, CNNs do not always outperform ANNs. A closer look at the plots reveals that this seems to be the case mostly for weekend data (Figs 9 and 10), whereas in weekdays (Figs 11 and 12) CNNs do perform significantly better than ANNs. The reason for this might be the fact that the employed time series, coming from the core network of an Internet Service Provider, are more structured during weekdays than weekends. The absence of such structure would hamper the ability of any model, no matter how sophisticated, to improve upon the performance exhibited by a simple ANN. This is the reason that on weekends, even though the CNN-c still performs slightly better, and ANNs and CNNs seem to be able to capture the structure of the data at wide time scales better than the naive approach, the margin is small and thus we cannot make conclusive statements about its superiority. On weekdays, however, the margin of superiority of the CNN-c model is much more significant and extremely unlikely to be due to pure randomness. Therefore, the use of CNNs with multiresolution seems clearly beneficial, at least in the case of weekday data.

#### 5.3.1 The effect of context

To assess whether the multiresolution input helps improve the quality of the forecasts we conducted three additional experiments. In the first experiment, we trained and evaluated neural networks without the added context -that is, just with 60 consecutive past observations as input- on the same training-test data pairs as above and compared with CNNs with multiresolution layout. In the second experiment, we trained and evaluated ANNs and CNNs with 240 past observations as input, without a multiresolution layout. This way, we give the models access to the same information as the ones trained with multiresolution input. Finally, in the third experiment we trained CNNs with only a 60-dimensional input with an exponential degree of 2 (i.e., 240 observations aggregated every 40 steps) in order to explore the relevance of high exponential context degrees for coarse-grained predictions.

**Experiment 1**. We trained and evaluated neural networks without the added context using 60 consecutive past observations as input and compared with CNNs with multiresolution layout and ARIMA models. Tables A through F in S1 File show the results obtained by ANNs and CNNs. Relative errors with respect to the variance and mean absolute deviation of each period (that is, $\frac{MSE}{\sigma^{2}}$ and $\frac{MAE}{MAD}$) are shown in Tables G through L in S1 File. These results are compared to the errors obtained by the ARIMA models. Tables A, C and E in S1 File show the results for the networks without context (that is, with an input consisting simply of the last 60 observations), denoted by ANN and CNN. Tables B, D and F in S1 File show the results obtained by the networks using context, that is, with exponential context degrees of 0, 1 and 2, denoted by ANN-c and CNN-c. Table 4, in turn, shows the relative improvement of each model with respect to the naive baseline, measured as $1-\frac{\text{model}\;\text{error}}{\text{naive}\;\text{error}}$. We report the average error over data partitions for each task, (i.e. each number of aggregated steps ahead), for both MSE and MAE. At the one-second scale, all models seem to perform similarly. However, as the aggregation scale increases, convolutional networks are clearly superior, especially when incorporating multiresolution input. This is consistent with intuition, as one expects coarse events at a large scale to be correlated with coarse events relatively far back in the past. Absolute error values in Tables B, D and F, and relative ones in Tables H, J and L in S1 File show that the edge of CNNs over the rest of the models is significant.

**Table 4:** Average percentage of improvement of each model over naive approach.

Steps ahead	Error	ANN	CNN	ANN-c	CNN-c	ARIMA(0, 1, 0)	ARIMA(1, 1, 0)
1	MAE	0.32	1.00	0.76	0.62	0.04	0.12
2		4.12	5.04	3.23	4.68	3.79	4.36
4		9.62	10.80	8.99	11.30	7.83	7.98
8		8.04	9.25	7.60	8.70	4.31	4.84
16		8.51	8.65	7.57	8.82	1.32	1.71
32		9.28	10.77	10.47	12.85	0.01	0.34
64		12.52	14.89	17.23	21.45	1.43	1.74
Avg.		5.24	6.04	5.43	6.72	1.57	1.67
1	MSE	4.83	6.65	3.20	5.33	4.46	11.43
2		13.87	15.18	13.82	15.37	2.09	2.24
4		23.34	23.83	22.68	25.60	17.08	15.76
8		18.38	17.65	18.39	18.81	8.79	11.90
16		19.50	18.15	20.87	20.03	1.33	4.16
32		25.77	18.37	29.44	28.71	0.52	0.11
64		34.32	33.56	40.65	45.90	2.91	5.16
Avg.		14.00	13.34	14.90	15.97	3.13	1.28

**Experiment 2**. We trained and evaluated ANNs and CNNs models using the 240 consecutive past observations as input (i.e., without a multiresolution layout) to give the models access to the same information as the ones trained with multiresolution input. The results are shown in Table M in S1 File. This experiment was only carried out for forecasts 32 and 64 steps ahead, where model sophistication has a more noticeable impact on forecast accuracy. In the case of weekend data, the CNN with multiresolution is superior, although by a smaller margin. However, In the case of weekday data, the CNN-c model is clearly superior to the rest of the approaches. This results suggest that whenever the data are sufficiently structured, the multiresolution input in combination with the CNN is clearly helpful in improving the quality of the forecasts. This is consistent with our previous observations in which weekday traffic appears to be more structured than that of weekends, and so more complex models can exploit these apparent regularities to make better forecasts. We can therefore conclude that the multiresolution approach indeed shows better performance in general. In addition, it should be noted that increasing the dimensionality of the input data is not always necessarily helpful for the learning procedure. Our approach to multiresolution context modeling, however, seems to offer a good trade-off between additional information and statistical efficiency.

**Experiment 3**. Finally, it could be argued that only the input data with a high exponential context degree is relevant for coarse-grained predictions. For instance, for predicting 64 aggregated steps ahead, one might think that taking a 60-dimensional input with an exponential context degree of 2 (that is, 240 observations aggregated every 4 steps) could be enough. To assess this we trained CNNs in this fashion (that is, only with exponential context degree of 2 as input) for all partitions at 32 and 64 aggregated steps. Table 5 shows the results with granularities of 32 and 64 aggregated steps ahead (SA). In the case of 32 aggregated steps ahead, it can be seen that the performance of these networks is generally slightly worse than that of the CNN-c models (particularly in the case of MAE), that is, the ones with exponential context degrees of 0, 1 and 2. This supports the hypothesis that multiple convolution channels representing previous observations at different levels of resolution are helpful for improving the quality of the forecasts. In the case of 64 aggregated steps ahead, the superiority is not clear. The difference between both cases could be related to the fact that high-resolution input (that is, with an exponential degree of 0) might be more relevant to 32-step forecasts. However, it would be interesting to assess the usefulness of the multiresolution input with more experiments and using larger timescales as input.

**Table 5:** Errors obtained by convolutional neural networks with single resolution input.

Partition	SA	CNN-context_degree:2	
		MSE	MAE
P1	32	40118.02	129.01
P2		41068.72	98.38
P3		16215.16	88.10
P4		14751.86	78.73
P5		43957.34	126.39
P6		133141.97	209.99
P7		67727.29	170.55
P8		57288.46	147.50
P9		52363.79	140.19
P10		135754.95	211.87
P1	64	39980.17	142.40
P2		26743.76	106.82
P3		18202.85	98.75
P4		16545.59	88.68
P5		50503.30	151.79
P6		107604.68	218.14
P7		67874.94	182.11
P8		54931.68	165.23
P9		48656.83	151.79
P10		119045.86	233.71

### 5.4 Durability of the models

The previous experiments have shown that ANNs and CNNs can provide significantly better forecasts than ARIMA as the time scale increases. However, in all tests (except for partitions P5 and P10 that were trained with the last month data) we trained the models on the most recent period available prior to the test data. An equivalent approach in a real-world scenario would require the user to retrain the models every month. We ran an additional set of experiments to try to determine whether a model trained on data from one month would remain robust enough after a few months have passed. Specifically, we trained models for the different time scales on data from February and March and tested them on the rest of the months.

**Fig 13:**
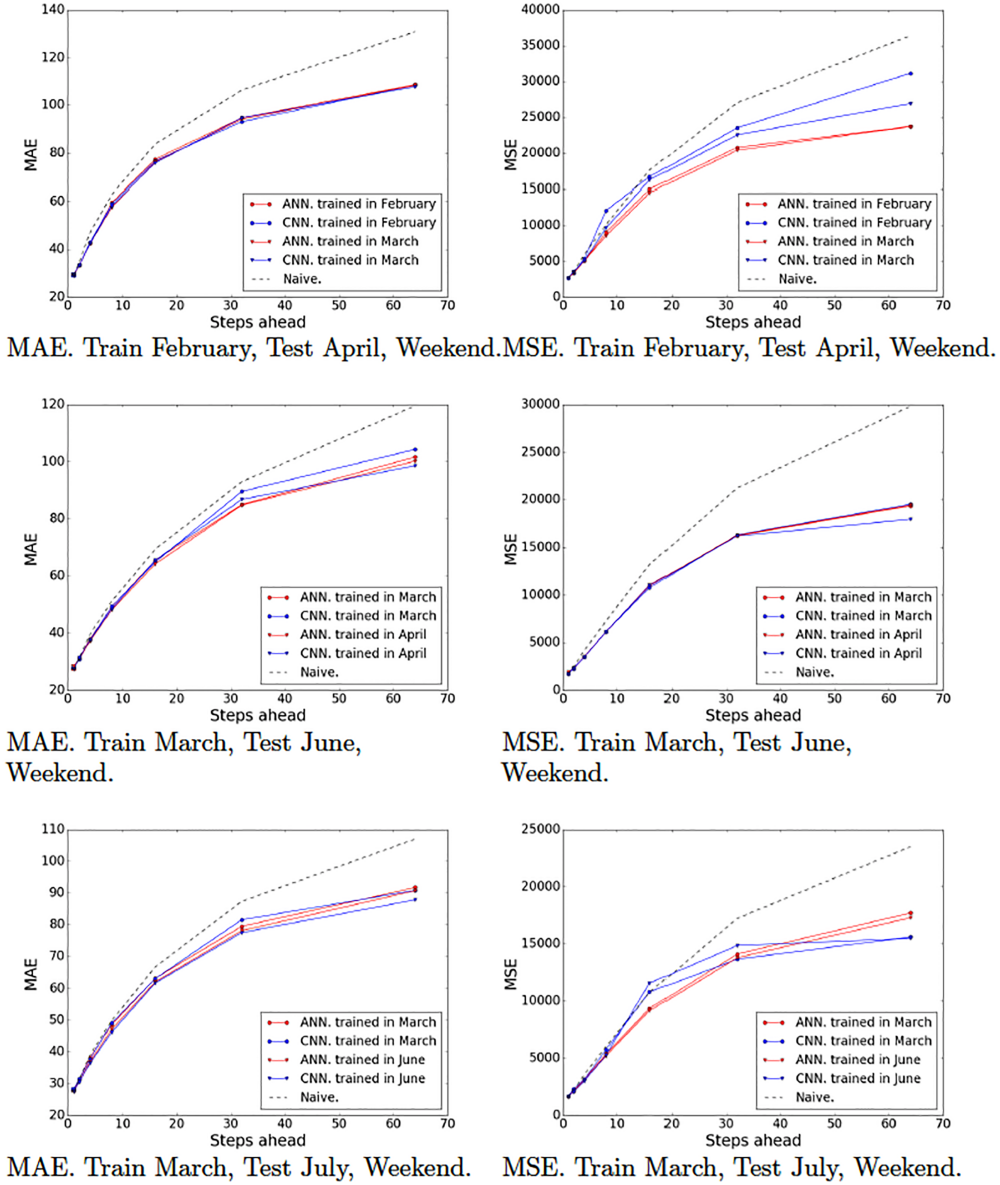
Model durability tests. Naive, ARIMA and ANN and CNN with multiresolution context. Weekends.

**Fig 14:**
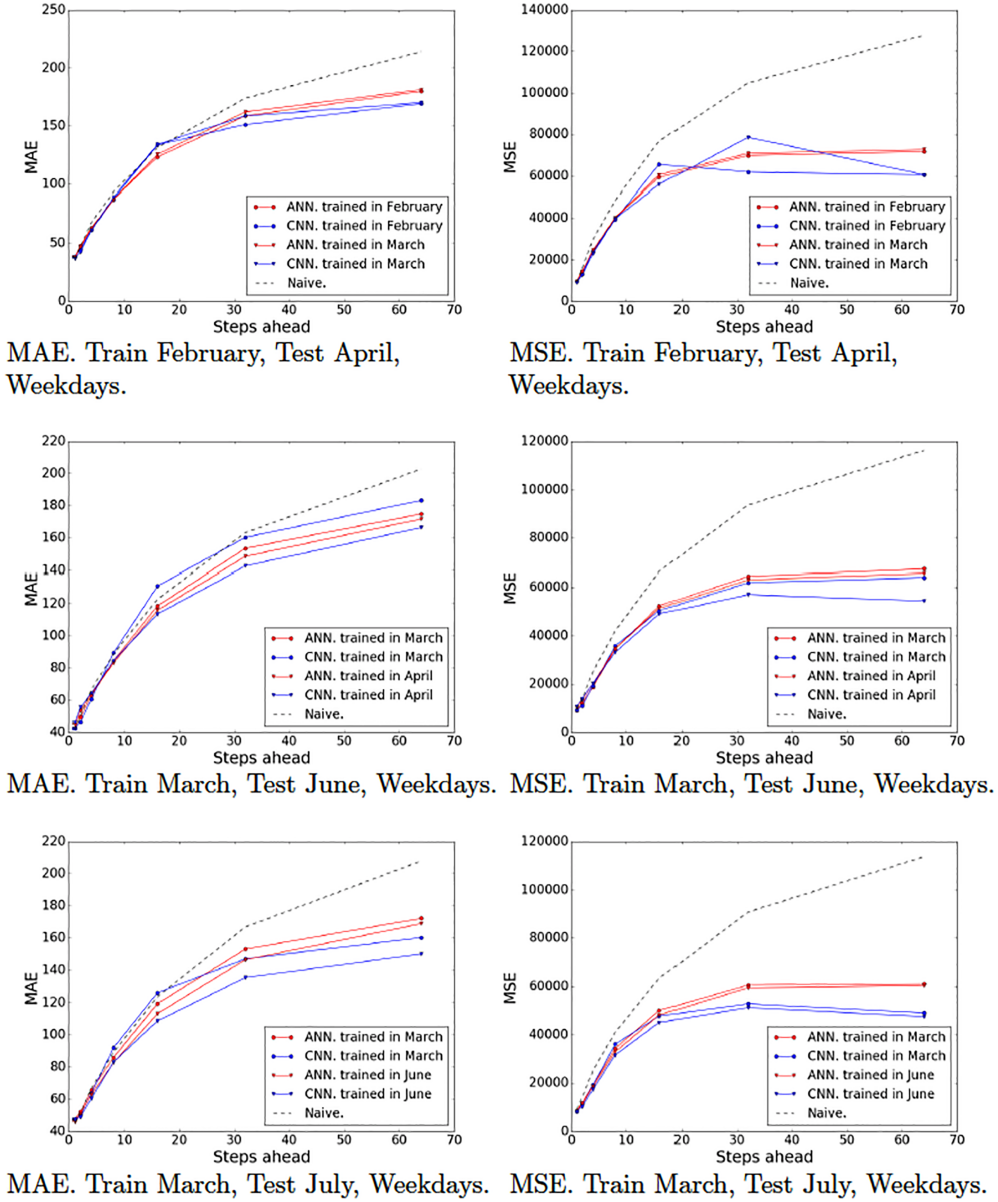
Model durability tests. Naive, ARIMA and ANN and CNN with multiresolution context. Weekdays.

Figs 13 and 14 show the error plots obtained with models trained on different periods. In general, the models trained on more recent periods seem to perform slightly better than those trained on earlier months. This is consistent with the intuition that the dynamics of traffic change slowly over time, which makes it convenient to periodically retrain the models. In some cases, the trained models did overfit severely and produced bad results (e.g., April weekdays, 32 aggregated steps). This was solved again by retraining with stronger regularization penalties (*λ* = 2.477*e* − 06 for convolutional layers and *λ* = 3.619*e* − 06 for fully-connected layers).

#### 5.4.1 Prediction speed

Since short-term time series forecasting is a real-time task, the time necessary to produce a prediction with each of the models is vital. We measured the time required by the different models to produce a forecast. All the models were fitted and evaluated on a 12-core Intel Xeon CPU at 2.60GHz with a Geforce GTX 1080 8GB GDDR5X GPU card. The ARIMA models took about 0.16 seconds for each produced value and both ANNs and CNNs required between 1e-5 and 1e-6 for each prediction. It should be noted that a more efficient approach could be adopted for the ARIMA models, like fitting a model a priori and using it to make predictions on new data. However, as the obtained results were not quite good, we took the approach that we deemed best suited for the task at hand in terms of the quality of the forecasts.

#### 5.4.2 Remarks

Our remarks after running these experiments can be summarized as follows.

- First of all, a discussion on the cost of training neural networks is necessary. Other approaches, while probably less effective, would have taken seconds to train, whereas each of our networks took about one hour using proper -though admittedly not the most powerful available- hardware. Initially, it is time consuming to validate hypotheses regarding the hyperparameters and the network architecture. However, once the networks are properly tuned, they seem to exhibit robust behaviour when trained once a month, and therefore one or even a few hours of training should be admissible.
- Model complexity, if not properly regularized, can be very risky, as it can occasionally produce large errors. It is therefore essential to extensively validate hyperparameter choices.
- Convolutional layers seem to help improve forecasts when the input data are sufficiently structured, as can be observed in the results on weekday data in comparison to weekends.

## 6 Conclusions & future work

We have investigated the problem of forecasting short-term changes in data center network traffic load. We discovered that the behaviour of network traffic at the one-second scale is highly chaotic. So far it has been unclear whether any meaningful structure could be exploited to make reliable forecasts. Moreover, our analysis corresponding to the ARIMA model in subsection 5.2.2 reveals the lack of structure, which clearly shows that the problem is hard to solve from a traditional time-series-analysis perspective.

To the best of our knowledge, there are no previous attempts to solve this problem reported in the literature. Therefore, we have employed ARIMA as a baseline method to compare the effectiveness of our contribution.

In this context, we propose the use of convolutional neural networks (CNNs) -a type of neural network that can exploit the temporal nature of the data efficiently- in order to verify whether there exist highly non-linear regularities that can be learned by models more complex than traditional time-series-analysis methods. Additionally, we investigated different approaches to improve the quality of the forecasts -multiresolution context and coarse-grained forecasts. The main conclusion of this work is that the improvements exhibited by our CNN models with multiresolution input confirm that the highly non-linear regularities present in network traffic load can be captured and learned by CNNs. Moreover, the proposed CNN models can be trained monthly in a reasonable amount of time showing a quite long durability and can produce forecasts fast enough for real-time use. These properties encourage the utilization of CNNs for use in real-time data center scenarios.

We tested the ability of the models to forecast the number of active TCP flows in a network traffic trace collected at the core network of a medium-sized Spanish ISP that processes about 18.7 million TCP flows per day on average. The trace was taken from a period spanning 5 months, totalling 70 days of traffic at the one-second resolution. We aggregated and transformed this trace in a time series representing the number of active TCP flows (user sessions) per second, totalling over 6 millions of data points. The short-term behavior of these data (one-second resolution) is very noisy making very short-term forecasts seemingly impossible to improve with respect to naive methods, even using state-of-the-art approaches. We therefore run experiments on increased time scales to determine if any improvements could be obtained. It should be noted that part of the noise present in the analyzed data might not be completely random and could perhaps be explained via exogenous variables related to the services and protocols running on the network (e.g. CPU and memory usage, or inbound and outbound network packets of the corresponding virtual machines). In the future it would be interesting to analyze whether a richer time series with this information could help to improve the quality of the forecasts.

We have shown that ANNs and especially CNNs, can make forecasts with a mean absolute error significantly below the mean absolute deviation of the differenced data at time scales of 64 seconds and below (i.e., our errors are smaller than the intrinsic variability of the data). Simple ARIMA models, however, do not seem to obtain better results than a naive approach. This suggests that the time series exhibits meaningful non-linear structure from the 16-second scale and above that can be captured by ANN and CNN models. Certain problems that arise in cloud and data center infrastructure, such as resource placement, require anticipatory decisions to be made within 60–120 seconds approximately. Therefore, our approach could prove useful for this purpose.

CNN models outperform both ARIMA and ANNs by an increasingly significant margin as the time scale increases, in both mean squared error and mean absolute error. Additionally, our efficient approach to context modeling via multiresolution input seems to enable promising performance improvements as well above the 16-second scale. This suggests that the time series exhibits long-term non-linear correlations that can be exploited for short-term forecasting. Moreover, the multi-resolution approach can be exploited efficiently using the multiple channels of a conventional convolutional neural network architecture.

A slight drawback of ANN and CNN models is the training procedure, which is generally slower than other methods (e.g. random forests for regression). However, modern GPU cards help to accelerate the training phase. Our proposed neural networks were trained in about an hour on a moderately powerful GPU, which is feasible if the models are to be retrained monthly or even weekly. Moreover, the trained models exhibit robust performance and durability. In addition, predictions can be made in about 1e-5 seconds, as opposed to the 0.16 seconds that our ARIMA-based approach required. These times jointly with the observed durability of our CNN models, make these models suitable for use in the real-time scenarios that are likely to arise in data center management.

In the future we plan to take additional steps to further improve the quality of the forecasts. First, we plan to test different scales of multiresolution input to incorporate more context for medium-term forecasts. It would also be interesting to enrich the present time series with exogenous variables such as the services and protocols running on the infrastructure and the topology of the network. Finally, we will attempt to evaluate whether these methods can be used on the actuation side to improve the efficiency of cloud infrastructure management tasks like resource scaling. The promising results obtained with convolutional networks motivate further research in this direction. In particular, it would be interesting to run experiments with deeper models, as this strategy has been successful in various application domains. It would also be interesting to evaluate the performance of recurrent neural networks and in particular long short term memory units, which while less efficient than convolutional networks, are particularly well suited to time series settings. This motivates a particularly compelling research question. Deep networks have replaced and outperformed the manual feature extraction procedures based on hand-crafted filters or signal transforms traditionally used by domain experts for computer vision and speech recognition. Traditional approaches for time series analysis and forecasting rely on a set of preprocessing techniques, such as identification of stationarity and seasonality, detrending, etc. Can deep models replace these methods and successfully analyse raw time series data for forecasting?

## Supporting information

S1 File Detailed forecasting results. Tables containing detailed results of the experiments.
(PDF)
