# Supplementary material for: Forecasting short-term data center network traffic load with convolutional neural networks
Source: PLoS One. 2018 Feb 6;13(2):e0191939. doi: 10.1371/journal.pone.0191939 (PMC5800645; doi:10.1371/journal.pone.0191939)
Supplement: S1 File — Tables containing detailed results of the experiments. (PDF) [file pone.0191939.s001.pdf]

Forecasting short-term data center network traffic load with convolutional neural networks

Supplementary file S1: Detailed forecasting results

Table A. Errors obtained by artificial and convolutional neural networks without context compared with ARIMA.

Partition	SA	ANN		CNN		ARIMA(0,1,0)		ARIMA(1,1,0)	
		MSE	MAE	MSE	MAE	MSE	MAE	MSE	MAE
P1	1	3513.42	37.28	3423.03	37.25	3646.82	38.02	3655.38	37.60
P2		2667.16	29.17	2756.53	29.20	2688.05	29.28	2790.12	29.31
P3		1745.76	27.54	1790.56	27.95	1791.42	27.42	**1649.04**	**27.01**
P4		1620.14	**27.44**	1660.07	27.56	1676.16	27.59	1684.06	27.67
P5		3170.70	33.17	3172.53	33.20	3378.25	34.04	3364.00	33.48
P6		9872.23	46.00	**9762.57**	**45.83**	11959.29	48.94	12607.12	48.58
P7		9426.70	36.77	8629.30	36.33	7503.58	**35.64**	**6574.82**	36.13
P8		10274.08	44.22	10744.80	44.74	**7883.89**	**42.11**	24382.45	43.17
P9		8295.98	45.76	**7463.21**	**44.13**	8016.71	44.56	8652.42	45.44
P10		12221.88	49.18	**10696.49**	**46.65**	14915.08	50.94	13970.17	50.71
P1	2	5062.31	43.68	4728.43	42.97	5164.91	43.91	**4459.59**	42.77
P2		3380.93	33.22	3541.51	**33.12**	4377.09	33.43	5080.94	34.34
P3		2313.02	31.47	2289.11	30.88	2214.07	**30.66**	**2196.13**	30.77
P4		2072.45	30.69	2082.88	30.73	2162.32	31.11	2171.86	31.10
P5		4233.97	37.20	**4113.65**	37.07	4560.32	38.05	4246.87	38.01
P6		15808.34	54.22	15642.97	54.21	20109.33	58.51	17988.46	56.93
P7		13741.09	44.29	13588.52	43.97	12178.01	44.40	**11250.91**	44.22
P8		13693.40	52.85	**13342.12**	50.33	21361.70	49.32	30979.19	**48.82**
P9		11161.15	50.99	10936.41	52.86	12408.73	50.44	12236.71	50.46
P10		18892.24	58.03	**18148.52**	**55.99**	21439.46	59.45	18899.36	58.08
P1	4	8223.04	55.41	8168.20	55.43	8682.34	56.79	8798.23	56.66
P2		5077.79	**42.37**	5905.84	42.53	5115.16	43.04	6859.47	43.48
P3		3521.06	38.02	3613.70	38.64	3784.06	38.17	**3287.03**	**37.19**
P4		3048.00	36.73	3023.03	36.64	3132.89	37.52	3270.78	37.75
P5		6823.83	45.49	6359.61	**43.90**	6843.03	46.44	6911.17	46.59
P6		30292.68	73.57	29360.64	71.26	39726.32	77.32	37236.12	76.06
P7		23612.22	62.60	23691.10	60.92	25855.01	61.61	**22453.76**	**60.76**
P8		20547.51	65.24	**18938.04**	62.68	20174.40	**61.62**	20008.79	62.47
P9		18113.68	61.27	**17343.49**	**59.62**	20389.05	62.31	22079.10	63.09
P10		34752.11	70.45	32933.72	71.43	43125.96	80.16	43252.53	79.83

Table B. Errors obtained by artificial and convolutional neural networks with context compared with ARIMA.

Partition	SA	ANN-c		CNN-c		ARIMA(0,1,0)		ARIMA(1,1,0)	
		MSE	MAE	MSE	MAE	MSE	MAE	MSE	MAE
P1	1	3564.33	37.42	**3417.54**	**36.76**	3646.82	38.02	3655.38	37.60
P2		**2660.52**	**29.11**	2689.66	29.61	2688.05	29.28	2790.12	29.31
P3		1919.64	28.40	1741.94	27.50	1791.42	27.42	**1649.04**	**27.01**
P4		**1617.87**	27.47	1649.32	27.62	1676.16	27.59	1684.06	27.67
P5		3167.69	33.19	**3041.83**	**32.83**	3378.25	34.04	3364.00	33.48
P6		10188.60	47.26	10176.94	47.98	11959.29	48.94	12607.12	48.58
P7		9568.65	37.74	9072.21	36.90	7503.58	**35.64**	**6574.82**	36.13
P8		10246.17	44.69	10744.47	46.17	**7883.89**	**42.11**	24382.45	43.17
P9		8377.00	46.02	8277.77	46.93	8016.71	44.56	8652.42	45.44
P10		12328.69	49.39	11647.12	48.25	14915.08	50.94	13970.17	50.71
P1	2	4838.23	42.71	4752.48	**42.61**	5164.91	43.91	**4459.59**	42.77
P2		**3362.04**	33.34	3548.62	33.31	4377.09	33.43	5080.94	34.34
P3		2297.39	31.08	2343.40	31.44	2214.07	**30.66**	**2196.13**	30.77
P4		2072.89	30.66	**2053.40**	**30.57**	2162.32	31.11	2171.86	31.10
P5		4220.90	37.29	4158.95	**36.97**	4560.32	38.05	4246.87	38.01
P6		16369.79	57.13	**15411.57**	**53.61**	20109.33	58.51	17988.46	56.93
P7		14190.80	46.70	12638.19	**42.53**	12178.01	44.40	11250.91	44.22
P8		13507.41	53.47	13922.93	55.75	21361.70	49.32	30979.19	**48.82**
P9		11347.47	50.98	**10356.77**	**49.13**	12408.73	50.44	12236.71	50.46
P10		19095.78	58.60	18823.29	58.07	21439.46	59.45	18899.36	58.08
P1	4	8061.59	**54.58**	**8000.28**	54.78	8682.34	56.79	8798.23	56.66
P2		**5048.15**	42.44	5079.63	42.49	5115.16	43.04	6859.47	43.48
P3		3539.05	37.21	3490.34	37.58	3784.06	38.17	**3287.03**	**37.19**
P4		3052.90	36.88	**3015.56**	**36.39**	3132.89	37.52	3270.78	37.75
P5		6764.95	45.62	**6330.10**	43.97	6843.03	46.44	6911.17	46.59
P6		30604.72	73.33	**28870.48**	**68.18**	39726.32	77.32	37236.12	76.06
P7		24805.61	62.72	22924.02	61.16	25855.01	61.61	**22453.76**	**60.76**
P8		20325.01	64.11	20340.48	64.48	20174.40	**61.62**	20008.79	62.47
P9		18613.63	63.00	17356.89	60.45	20389.05	62.31	22079.10	63.09
P10		36378.62	77.58	**32454.39**	**70.12**	43125.96	80.16	43252.53	79.83

Table C. Errors obtained by artificial and convolutional neural networks without context compared with ARIMA.

Partition	SA	ANN		CNN		ARIMA(0,1,0)		ARIMA(1,1,0)	
		MSE	MAE	MSE	MAE	MSE	MAE	MSE	MAE
P1	8	**15919.89**	76.93	16160.31	76.52	16708.92	80.27	17502.78	80.06
P2		8771.88	58.21	10263.52	**57.12**	10176.10	59.62	**8119.16**	59.31
P3		6309.57	48.28	6196.50	49.01	6496.65	49.79	6761.55	50.48
P4		**5202.10**	46.99	5217.42	46.96	5265.02	48.23	5663.03	48.87
P5		13442.44	61.32	**12992.65**	**60.33**	13816.52	64.05	14061.07	64.83
P6		64419.99	103.86	**60612.79**	**98.35**	85308.32	116.45	81711.36	113.55
P7		**39465.94**	90.82	43868.28	88.31	43693.33	88.24	39790.93	**87.09**
P8		34397.06	83.53	34069.60	86.13	38311.36	85.36	38034.09	85.28
P9		32926.24	79.52	**31395.80**	**79.45**	42531.52	88.13	37518.45	85.69
P10		74068.67	111.21	**69122.97**	**104.63**	82110.29	114.24	80419.43	111.84
P1	16	28846.83	104.12	29015.14	104.68	33553.29	113.28	31459.33	111.36
P2		15112.13	77.47	19305.25	78.06	17525.57	82.33	16651.74	81.87
P3		11339.94	65.24	11566.63	66.11	12297.17	67.93	13064.80	69.13
P4		9509.73	**61.37**	9491.30	62.50	10577.63	66.44	10365.69	66.26
P5		26527.08	86.36	25445.89	**85.43**	30596.32	93.45	28657.62	93.78
P6		113399.09	**154.92**	111851.04	155.49	163462.15	175.95	149501.37	170.83
P7		59455.47	130.33	59087.43	128.17	75747.87	129.30	79892.81	132.35
P8		50721.02	112.61	50204.73	**111.91**	67125.45	121.93	65841.27	122.83
P9		51853.29	115.26	47636.10	109.45	66382.94	124.68	62063.18	122.14
P10		115625.49	**150.09**	115361.66	153.74	155859.34	173.31	150667.77	170.28

Table D. Errors obtained by artificial and convolutional neural networks with context compared with ARIMA.

Partition	SA	ANN-c		CNN-c		ARIMA(0,1,0)		ARIMA(1,1,0)	
		MSE	MAE	MSE	MAE	MSE	MAE	MSE	MAE
P1	8	16401.93	78.21	16330.96	**76.14**	16708.92	80.27	17502.78	80.06
P2		8583.26	57.59	9687.01	58.09	10176.10	59.62	**8119.16**	59.31
P3		**6132.47**	**48.15**	6146.74	48.39	6496.65	49.79	6761.55	50.48
P4		5210.13	47.16	5271.90	**46.22**	5265.02	48.23	5663.03	48.87
P5		13248.28	62.13	13301.80	61.41	13816.52	64.05	14061.07	64.83
P6		67540.82	108.38	61739.37	99.93	85308.32	116.45	81711.36	113.55
P7		40358.98	87.26	39735.17	89.35	43693.33	88.24	39790.93	**87.09**
P8		34633.82	**83.11**	**33142.90**	84.57	38311.36	85.36	38034.09	85.28
P9		32916.69	82.62	31574.09	82.99	42531.52	88.13	37518.45	85.69
P10		71376.83	110.89	69342.00	105.67	82110.29	114.24	80419.43	111.84
P1	16	**27662.28**	102.61	28134.91	**101.73**	33553.29	113.28	31459.33	111.36
P2		**14521.27**	76.25	16246.81	**75.81**	17525.57	82.33	16651.74	81.87
P3		11075.94	**64.20**	**11027.11**	65.57	12297.17	67.93	13064.80	69.13
P4		**9164.43**	62.04	11578.10	61.65	10577.63	66.44	10365.69	66.26
P5		26118.37	89.22	**25430.75**	86.94	30596.32	93.45	28657.62	93.78
P6		118086.65	162.02	**108309.95**	155.58	163462.15	175.95	149501.37	170.83
P7		60628.43	**125.60**	**56055.67**	134.22	75747.87	129.30	79892.81	132.35
P8		51383.78	115.81	**49062.69**	113.42	67125.45	121.93	65841.27	122.83
P9		48204.25	113.12	**45092.99**	**108.55**	66382.94	124.68	62063.18	122.14
P10		115737.32	164.98	**111810.37**	151.67	155859.34	173.31	150667.77	170.28

Table E. Errors obtained by artificial and convolutional neural networks without context compared with ARIMA.

Partition	SA	ANN		CNN		ARIMA(0,1,0)		ARIMA(1,1,0)	
		MSE	MAE	MSE	MAE	MSE	MAE	MSE	MAE
P1	32	39303.11	126.97	40662.1	129.9	50729.56	145.12	50418.40	145.73
P2		22661.0	95.4	49707.4	96.4	26670.53	106.15	26281.36	106.37
P3		17562.7	87.8	17092.6	86.0	21193.72	93.36	21420.73	93.83
P4		13847.3	78.9	**13764.1**	78.5	17239.94	88.00	17182.55	88.02
P5		43610.1	**120.0**	44209.7	122.7	53844.85	136.64	54197.14	136.25
P6		148592.1	218.6	141082.1	207.5	250854.15	254.81	252693.50	254.61
P7		69397.0	170.7	66713.7	166.7	103434.48	172.18	112711.39	179.47
P8		61423.2	145.7	60361.7	144.0	90422.95	161.35	89081.92	161.27
P9		68696.3	153.7	60321.0	148.7	92446.43	169.50	92353.01	168.85
P10		166932.5	227.9	141253.3	211.4	234465.27	247.05	232158.50	243.82
P1	64	46273.9	152.1	48706.0	155.4	69989.86	183.21	71062.25	183.76
P2		26752.4	114.9	41887.7	117.9	38862.89	134.10	38596.21	132.71
P3		22559.3	108.5	21875.7	108.3	30467.07	120.73	31070.71	121.73
P4		17407.8	93.7	17045.1	92.9	24029.77	108.69	23993.74	108.81
P5		55008.9	154.4	55829.7	156.4	78230.03	183.02	78867.83	183.64
P6		141094.3	255.1	129868.3	247.6	311520.48	322.62	322248.17	324.84
P7		71715.9	190.6	75276.1	188.0	131332.17	216.25	143523.13	220.23
P8		67395.4	176.9	67131.9	176.9	119074.34	206.20	124679.27	206.29
P9		78981.9	190.5	58019.5	165.2	116845.48	211.02	116800.87	211.07
P10		181778.9	286.2	139676.4	247.8	291247.42	318.22	294198.03	318.06

Table F. Errors obtained by artificial and convolutional neural networks with context compared with ARIMA.

Partition	SA	ANN-c		CNN-c		ARIMA(0,1,0)		ARIMA(1,1,0)	
		MSE	MAE	MSE	MAE	MSE	MAE	MSE	MAE
P1	32	37903.5	**124.8**	**37140.84**	124.94	50729.56	145.12	50418.40	145.73
P2		**20371.5**	**94.0**	22188.24	94.90	26670.53	106.15	26281.36	106.37
P3		16281.7	**84.7**	**16066.56**	84.75	21193.72	93.36	21420.73	93.83
P4		13789.9	78.1	19234.39	**77.95**	17239.94	88.00	17182.55	88.02
P5		**42350.8**	126.5	43417.93	122.58	53844.85	136.64	54197.14	136.25
P6		149728.9	220.2	**126737.77**	**204.19**	250854.15	254.81	252693.50	254.61
P7		70871.1	162.2	**61881.03**	**158.73**	103434.48	172.18	112711.39	179.47
P8		63049.0	148.7	**56629.57**	**141.98**	90422.95	161.35	89081.92	161.27
P9		59450.4	146.6	**55556.73**	**140.83**	92446.43	169.50	92353.01	168.85
P10		143344.0	220.6	**139804.31**	**206.23**	234465.27	247.05	232158.50	243.82
P1	64	**41936.2**	143.9	43144.4	**142.6**	69989.86	183.21	71062.25	183.76
P2		**23682.4**	108.4	26903.4	**107.7**	38862.89	134.10	38596.21	132.71
P3		19451.8	100.2	**17961.5**	**98.5**	30467.07	120.73	31070.71	121.73
P4		17291.6	90.7	**15497.8**	**87.9**	24029.77	108.69	23993.74	108.81
P5		51812.7	156.7	**49347.6**	**150.5**	78230.03	183.02	78867.83	183.64
P6		139283.8	251.0	**107530.6**	**218.4**	311520.48	322.62	322248.17	324.84
P7		72754.7	181.4	**60620.6**	**170.2**	131332.17	216.25	143523.13	220.23
P8		65618.6	171.7	**54387.4**	**166.4**	119074.34	206.20	124679.27	206.29
P9		60333.1	169.0	**47512.8**	**150.2**	116845.48	211.02	116800.87	211.07
P10		137445.9	253.0	**119937.0**	**232.8**	291247.42	318.22	294198.03	318.06

Table G. Relative errors obtained by artificial and convolutional neural networks without context compared with ARIMA.

Partition	SA	ANN		CNN		ARIMA(0,1,0)		ARIMA(1,1,0)	
		$\frac{MSE}{\sigma^2}$	$\frac{MAE}{MAD}$	$\frac{MSE}{\sigma^2}$	$\frac{MAE}{MAD}$	$\frac{MSE}{\sigma^2}$	$\frac{MAE}{MAD}$	$\frac{MSE}{\sigma^2}$	$\frac{MAE}{MAD}$
P1	1	0.978	0.987	0.953	0.986	1.015	1.006	1.017	0.995
P2		1.004	0.998	1.038	0.999	1.012	1.002	1.050	1.003
P3		1.007	1.010	1.033	1.025	1.033	1.005	**0.951**	**0.990**
P4		0.985	**0.996**	1.010	1.001	1.019	1.002	1.024	1.005
P5		0.987	0.988	0.987	0.988	1.051	1.014	1.047	0.997
P6		0.768	0.939	**0.760**	**0.935**	0.931	0.999	0.981	0.991
P7		1.013	1.011	0.927	0.999	0.806	**0.980**	**0.706**	0.993
P8		1.046	1.060	1.094	1.073	**0.802**	**1.010**	2.482	1.035
P9		0.899	1.006	**0.809**	**0.970**	0.869	0.980	0.938	0.999
P10		0.839	0.974	**0.734**	**0.923**	1.023	1.008	0.959	1.004
P1	2	0.766	0.880	0.715	0.866	0.781	0.885	**0.675**	0.862
P2		0.872	0.883	0.914	**0.880**	1.129	0.889	1.311	0.913
P3		0.808	0.934	0.799	0.916	0.773	**0.910**	**0.767**	0.913
P4		0.818	0.914	0.822	0.915	0.854	0.926	0.858	0.926
P5		0.777	0.898	**0.755**	0.895	0.837	0.918	0.779	0.917
P6		0.537	0.801	0.531	0.800	0.683	0.864	0.611	0.841
P7		0.715	0.845	0.707	0.838	0.634	0.847	**0.586**	0.843
P8		0.845	0.975	**0.823**	0.928	1.318	0.910	1.912	**0.900**
P9		0.680	0.927	0.667	0.961	0.757	0.917	0.746	0.917
P10		0.595	0.847	**0.572**	**0.818**	0.675	0.868	0.595	0.848
P1	4	0.550	0.748	0.546	0.749	0.581	0.767	0.588	0.765
P2		0.634	**0.764**	0.738	0.767	0.639	0.776	0.857	0.784
P3		0.619	0.833	0.635	0.846	0.665	0.836	**0.577**	**0.815**
P4		0.643	0.820	0.638	0.818	0.661	0.837	0.690	0.842
P5		0.565	0.777	0.527	**0.750**	0.567	0.793	0.572	0.796
P6		0.410	0.700	0.397	0.678	0.538	0.735	0.504	0.723
P7		0.586	0.776	0.588	0.755	0.642	0.763	**0.557**	**0.753**
P8		0.594	0.836	**0.548**	0.803	0.584	**0.790**	0.579	0.801
P9		0.529	0.784	**0.506**	**0.763**	0.595	0.798	0.645	0.808
P10		0.460	0.677	0.436	0.686	0.571	0.770	0.573	0.767

Table H. Relative errors obtained by artificial and convolutional neural networks with context compared with ARIMA.

Partition	SA	ANN-c		CNN-c		ARIMA(0,1,0)		ARIMA(1,1,0)	
		$\frac{MSE}{\sigma^2}$	$\frac{MAE}{MAD}$	$\frac{MSE}{\sigma^2}$	$\frac{MAE}{MAD}$	$\frac{MSE}{\sigma^2}$	$\frac{MAE}{MAD}$	$\frac{MSE}{\sigma^2}$	$\frac{MAE}{MAD}$
P1	1	0.992	0.990	**0.951**	**0.973**	1.015	1.006	1.017	0.995
P2		**1.001**	**0.996**	1.012	1.013	1.012	1.002	1.050	1.003
P3		1.107	1.041	1.005	1.008	1.033	1.005	**0.951**	**0.990**
P4		**0.984**	0.997	1.003	1.003	1.019	1.002	1.024	1.005
P5		0.986	0.988	**0.947**	**0.977**	1.051	1.014	1.047	0.997
P6		0.793	0.964	0.792	0.979	0.931	0.999	0.981	0.991
P7		1.028	1.037	0.975	1.014	0.806	**0.980**	**0.706**	0.993
P8		1.043	1.072	1.094	1.107	**0.802**	**1.010**	2.482	1.035
P9		0.908	1.012	0.897	1.032	0.869	0.980	0.938	0.999
P10		0.846	0.978	0.799	0.955	1.023	1.008	0.959	1.004
P1	2	0.732	0.860	0.719	**0.858**	0.781	0.885	**0.675**	0.862
P2		**0.867**	0.886	0.916	0.885	1.129	0.889	1.311	0.913
P3		0.802	0.922	0.818	0.933	0.773	**0.910**	**0.767**	0.913
P4		0.818	0.913	**0.811**	**0.910**	0.854	0.926	0.858	0.926
P5		0.774	0.900	0.763	**0.892**	0.837	0.918	0.779	0.917
P6		0.556	0.843	**0.523**	**0.791**	0.683	0.864	0.611	0.841
P7		0.739	0.891	0.658	**0.811**	0.634	0.847	0.586	0.843
P8		0.833	0.986	0.859	1.028	1.318	0.910	1.912	**0.900**
P9		0.692	0.926	**0.631**	**0.893**	0.757	0.917	0.746	0.917
P10		0.602	0.856	0.593	0.848	0.675	0.868	0.595	0.848
P1	4	0.539	**0.737**	**0.535**	0.740	0.581	0.767	0.588	0.765
P2		**0.631**	0.765	0.634	0.766	0.639	0.776	0.857	0.784
P3		0.622	0.815	0.613	0.823	0.665	0.836	**0.577**	**0.815**
P4		0.644	0.823	**0.636**	**0.812**	0.661	0.837	0.690	0.842
P5		0.560	0.779	**0.524**	0.751	0.567	0.793	0.572	0.796
P6		0.414	0.697	**0.391**	**0.648**	0.538	0.735	0.504	0.723
P7		0.616	0.777	0.569	0.758	0.642	0.763	**0.557**	**0.753**
P8		0.588	0.822	0.588	0.826	0.584	**0.790**	0.579	0.801
P9		0.544	0.806	0.507	0.774	0.595	0.798	0.645	0.808
P10		0.481	0.745	**0.429**	**0.673**	0.571	0.770	0.573	0.767

Table I. Relative errors obtained by artificial and convolutional neural networks without context compared with ARIMA.

Partition	SA	ANN		CNN		ARIMA(0,1,0)		ARIMA(1,1,0)	
		$\frac{MSE}{\sigma^2}$	$\frac{MAE}{MAD}$	$\frac{MSE}{\sigma^2}$	$\frac{MAE}{MAD}$	$\frac{MSE}{\sigma^2}$	$\frac{MAE}{MAD}$	$\frac{MSE}{\sigma^2}$	$\frac{MAE}{MAD}$
P1	8	**0.500**	0.701	0.507	0.697	0.524	0.732	0.549	0.730
P2		0.544	0.724	0.637	**0.710**	0.632	0.741	**0.504**	0.737
P3		0.530	0.745	0.520	0.756	0.546	0.768	0.568	0.779
P4		**0.535**	0.750	0.537	0.750	0.541	0.770	0.582	0.780
P5		0.471	0.690	**0.455**	**0.679**	0.484	0.720	0.493	0.729
P6		0.404	0.624	**0.380**	**0.591**	0.535	0.700	0.513	0.682
P7		**0.542**	0.741	0.603	0.720	0.601	0.720	0.547	**0.710**
P8		0.550	0.736	0.545	0.759	0.613	0.753	0.608	0.752
P9		0.554	0.700	**0.529**	**0.699**	0.716	0.776	0.632	0.754
P10		0.497	0.690	**0.464**	**0.649**	0.551	0.708	0.540	0.693
P1	16	0.554	0.734	0.557	0.738	0.644	0.799	0.604	0.785
P2		0.565	0.744	0.722	0.750	0.655	0.791	0.623	0.787
P3		0.533	0.730	0.543	0.740	0.578	0.761	0.614	0.774
P4		0.555	**0.725**	0.554	0.738	0.618	0.785	0.605	0.783
P5		0.477	0.656	0.458	**0.649**	0.550	0.710	0.516	0.712
P6		0.442	**0.638**	0.436	0.640	0.638	0.724	0.583	0.703
P7		0.569	0.786	0.565	0.773	0.725	0.779	0.764	0.798
P8		0.550	0.728	0.544	**0.723**	0.728	0.788	0.714	0.794
P9		0.594	0.736	0.545	0.699	0.760	0.796	0.711	0.780
P10		0.502	**0.643**	0.501	0.659	0.677	0.743	0.654	0.730

Table J. Relative errors obtained by artificial and convolutional neural networks with context compared with ARIMA.

Partition	SA	ANN-c		CNN-c		ARIMA(0,1,0)		ARIMA(1,1,0)	
		$\frac{MSE}{\sigma^2}$	$\frac{MAE}{MAD}$	$\frac{MSE}{\sigma^2}$	$\frac{MAE}{MAD}$	$\frac{MSE}{\sigma^2}$	$\frac{MAE}{MAD}$	$\frac{MSE}{\sigma^2}$	$\frac{MAE}{MAD}$
P1	8	0.515	0.713	0.513	**0.694**	0.524	0.732	0.549	0.730
P2		0.533	0.716	0.601	0.722	0.632	0.741	**0.504**	0.737
P3		**0.515**	**0.743**	0.516	0.746	0.546	0.768	0.568	0.779
P4		0.536	0.753	0.542	**0.738**	0.541	0.770	0.582	0.780
P5		0.464	0.699	0.466	0.691	0.484	0.720	0.493	0.729
P6		0.424	0.651	0.387	0.600	0.535	0.700	0.513	0.682
P7		0.555	0.712	0.546	0.729	0.601	0.720	0.547	**0.710**
P8		0.554	**0.733**	**0.530**	0.746	0.613	0.753	0.608	0.752
P9		0.554	0.727	0.532	0.730	0.716	0.776	0.632	0.754
P10		0.479	0.688	0.465	0.655	0.551	0.708	0.540	0.693
P1	16	**0.531**	0.724	0.540	**0.718**	0.644	0.799	0.604	0.785
P2		**0.543**	0.733	0.607	**0.728**	0.655	0.791	0.623	0.787
P3		0.520	**0.719**	**0.518**	0.734	0.578	0.761	0.614	0.774
P4		**0.535**	0.733	0.676	0.728	0.618	0.785	0.605	0.783
P5		0.470	0.678	**0.457**	0.660	0.550	0.710	0.516	0.712
P6		0.460	0.667	**0.422**	0.640	0.638	0.724	0.583	0.703
P7		0.580	**0.757**	**0.536**	0.809	0.725	0.779	0.764	0.798
P8		0.557	0.749	**0.532**	0.733	0.728	0.788	0.714	0.794
P9		0.552	0.723	**0.516**	**0.693**	0.760	0.796	0.711	0.780
P10		0.502	0.707	**0.485**	0.650	0.677	0.743	0.654	0.730

Table K. Relative errors obtained by artificial and convolutional neural networks without context compared with ARIMA.

Partition	SA	ANN		CNN		ARIMA(0,1,0)		ARIMA(1,1,0)	
		$\frac{MSE}{\sigma^2}$	$\frac{MAE}{MAD}$	$\frac{MSE}{\sigma^2}$	$\frac{MAE}{MAD}$	$\frac{MSE}{\sigma^2}$	$\frac{MAE}{MAD}$	$\frac{MSE}{\sigma^2}$	$\frac{MAE}{MAD}$
P1	32	0.589	0.761	0.609	0.778	0.760	0.870	0.755	0.873
P2		0.666	0.780	1.462	0.789	0.784	0.868	0.773	0.870
P3		0.609	0.776	0.593	0.760	0.735	0.825	0.743	0.829
P4		0.623	0.777	**0.619**	0.773	0.776	0.866	0.773	0.867
P5		0.571	**0.697**	0.579	0.713	0.705	0.794	0.710	0.792
P6		0.542	0.723	0.514	0.686	0.915	0.843	0.922	0.842
P7		0.643	0.855	0.618	0.835	0.959	0.862	1.045	0.899
P8		0.604	0.771	0.593	0.762	0.889	0.854	0.876	0.853
P9		0.691	0.799	0.607	0.773	0.930	0.881	0.930	0.878
P10		0.657	0.775	0.556	0.719	0.924	0.840	0.915	0.829
P1	64	0.675	0.811	0.710	0.828	1.021	0.977	1.037	0.980
P2		0.728	0.830	1.141	0.852	1.058	0.969	1.051	0.959
P3		0.668	0.803	0.648	0.802	0.903	0.894	0.921	0.901
P4		0.683	0.827	0.669	0.820	0.944	0.959	0.942	0.960
P5		0.667	0.764	0.677	0.774	0.949	0.906	0.957	0.909
P6		0.582	0.760	0.536	0.737	1.286	0.961	1.330	0.967
P7		0.646	0.836	0.678	0.824	1.184	0.948	1.294	0.966
P8		0.657	0.813	0.655	0.813	1.162	0.948	1.216	0.948
P9		0.788	0.869	0.579	0.754	1.166	0.963	1.166	0.963
P10		0.772	0.848	0.593	0.734	1.237	0.943	1.249	0.942

Table L. Relative errors obtained by artificial and convolutional neural networks with context compared with ARIMA.

Partition	SA	ANN-c		CNN-c		ARIMA(0,1,0)		ARIMA(1,1,0)	
		$\frac{MSE}{\sigma^2}$	$\frac{MAE}{MAD}$	$\frac{MSE}{\sigma^2}$	$\frac{MAE}{MAD}$	$\frac{MSE}{\sigma^2}$	$\frac{MAE}{MAD}$	$\frac{MSE}{\sigma^2}$	$\frac{MAE}{MAD}$
P1	32	0.568	**0.748**	**0.556**	0.749	0.760	0.870	0.755	0.873
P2		**0.599**	**0.769**	0.652	0.776	0.784	0.868	0.773	0.870
P3		0.564	**0.748**	**0.557**	0.749	0.735	0.825	0.743	0.829
P4		0.620	0.769	0.865	**0.767**	0.776	0.866	0.773	0.867
P5		**0.555**	0.735	0.569	0.712	0.705	0.794	0.710	0.792
P6		0.546	0.728	**0.462**	**0.675**	0.915	0.843	0.922	0.842
P7		0.657	0.812	**0.574**	**0.795**	0.959	0.862	1.045	0.899
P8		0.620	0.787	**0.557**	**0.751**	0.889	0.854	0.876	0.853
P9		0.598	0.762	**0.559**	**0.732**	0.930	0.881	0.930	0.878
P10		0.564	0.750	**0.551**	**0.701**	0.924	0.840	0.915	0.829
P1	64	**0.612**	0.767	0.629	**0.760**	1.021	0.977	1.037	0.980
P2		**0.645**	0.783	0.733	**0.778**	1.058	0.969	1.051	0.959
P3		0.576	0.742	**0.532**	**0.729**	0.903	0.894	0.921	0.901
P4		0.679	0.800	**0.608**	**0.775**	0.944	0.959	0.942	0.960
P5		0.628	0.776	**0.598**	**0.745**	0.949	0.906	0.957	0.909
P6		0.575	0.747	**0.443**	**0.650**	1.286	0.961	1.330	0.967
P7		0.656	0.795	**0.546**	**0.746**	1.184	0.948	1.294	0.966
P8		0.640	0.789	**0.530**	**0.765**	1.162	0.948	1.216	0.948
P9		0.602	0.771	**0.474**	**0.685**	1.166	0.963	1.166	0.963
P10		0.583	0.749	**0.509**	**0.689**	1.237	0.943	1.249	0.942

Table M. Errors obtained by artificial and convolutional neural networks without using multi-resolution approach. The results using multiresolution are shown alongside for convenience.

Partition	SA	ANN (240 steps)		CNN (240 steps)		ANN-c		CNN-c	
		MSE	MAE	MSE	MAE	MSE	MAE	MSE	MAE
P1	32	37875.46	**124.66**	37728.26	126.02	37903.5	124.8	**37140.84**	124.94
P2		**20362.72**	94.39	20619.85	94.86	20371.5	**94.0**	22188.24	94.90
P3		16295.40	**83.52**	**15784.56**	86.02	16281.7	84.7	16066.56	84.75
P4		**13775.97**	78.29	13911.13	78.19	13789.9	78.1	19234.39	**77.95**
P5		42533.59	125.34	42786.33	126.14	**42350.8**	126.5	43417.93	**122.58**
P6		149708.72	223.64	150360.77	223.10	149728.9	220.2	**126737.77**	**204.19**
P7		70715.60	162.52	71433.58	163.77	70871.1	162.2	**61881.03**	**158.73**
P8		63065.46	148.42	**55599.26**	145.31	63049.0	148.7	56629.57	**141.98**
P9		59453.46	146.65	60059.83	147.64	59450.4	146.6	**55556.73**	**140.83**
P10		143417.00	220.80	143439.57	219.82	143344.0	220.6	**139804.31**	**206.23**
P1	64	**41878.83**	144.61	41989.93	143.80	41936.2	143.9	43144.4	**142.6**
P2		**23669.05**	108.69	23699.58	108.60	23682.4	108.4	26903.4	**107.7**
P3		19570.48	100.90	18147.38	98.94	19451.8	100.2	**17961.5**	**98.5**
P4		17309.97	90.58	17309.88	90.76	17291.6	90.7	**15497.8**	**87.9**
P5		50622.79	153.35	51364.22	155.60	51812.7	156.7	**49347.6**	**150.5**
P6		139661.51	251.74	139362.15	252.30	139283.8	251.0	**107530.6**	**218.4**
P7		72626.33	180.29	72911.81	182.06	72754.7	181.4	**60620.6**	**170.2**
P8		65476.85	171.02	54512.97	**162.56**	65618.6	171.7	**54387.4**	166.4
P9		60367.23	169.01	60632.37	169.27	60333.1	169.0	**47512.8**	**150.2**
P10		137412.50	253.51	138644.67	256.07	137445.9	253.0	**119937.0**	**232.8**
